# A density functional theory study of Fe(II)/Fe(III) distribution in single layer green rust: a cluster approach

**DOI:** 10.1186/s12932-021-00076-0

**Published:** 2021-06-11

**Authors:** Weichao Sun, Dominique J. Tobler, Martin P. Andersson

**Affiliations:** 1 Nano-Science Center, Department of Chemistry, University of Copenhagen, 2100 Copenhagen, Denmark; 2 Department of Plant and Environmental Sciences, University of Copenhagen, Thorvaldsensvej 40, 1871 Frederiksberg C, Denmark; 3 Department of Chemical and Biochemical Engineering, Technical University of Denmark, 2800 Kgs, Lyngby, Denmark

**Keywords:** Green Rust, Single Layer, Density Functional Theory, Fe(II)/Fe(III) Distribution, Spin Density, Cluster Approach

## Abstract

Green rust (GR) is a potentially important compound for the reduction of heavy metal and organic pollutants in subsurface environment because of its high Fe(II) content, but many details of the actual reaction mechanism are lacking. The reductive capacity distribution within GR is a key to understand how and where the redox reaction occurs and computational chemistry can provide more details about the electronic properties of green rust. We constructed three sizes of cluster models of single layer GR (i.e., without interlayer molecules or ions) and calculated the charge distribution of these structures using density functional theory. We found that the Fe(II) and Fe(III) are distributed unevenly in the single layer GR. Within a certain range of Fe(II)/Fe(III) ratios, the outer iron atoms behave more like Fe(III) and the inner iron atoms behave more like Fe(II). These findings indicate that the interior of GR is more reductive than the outer parts and will provide new information to understand the GR redox interactions.

**Supplementary Information:**

The online version contains supplementary material available at 10.1186/s12932-021-00076-0.

## Introduction

Green rusts (GR) are a family of Fe(II), Fe(III) layered double hydroxides (LDH) that frequently form in oxygen-poor, Fe(II)-rich soils and waters [1–5]. GRs are composed of positively charged Fe(II), Fe(III) hydroxide layers that alternate with hydrated interlayers containing anions (e.g. SO_4_^2−^, CO_3_^2−^, Cl,^−^ etc.) and occasionally cations for charge compensation. GRs are represented by the general formula [Fe^II^_(1-*x*)_Fe^III^*x*(OH)_2_]^*x*+^[(*x*/*n*)A^*n*−^,mH_2_O]^*x*−^, where *x* represents the molar fraction of the ferric ion that usually ranges from 0.25 to 0.33 and A^n−^ denotes intercalated anions [2, 6–8]. GRs are classified into two types based on the anion they intercalate: [1] GR type 1 has a rhombohedral unit cell and intercalates planar or monatomic anions (e.g., Cl^−^, CO_3_^2−^) [9, 10]. GR type 2 has a hexagonal unit cell and intercalates three-dimensional anions (e.g., SO_4_^2−^) [11].

GRs have been widely investigated for removal of organic and inorganic contaminants from waters and soils [12–27] (e.g., SeO_4_^2−^ [13], U^6+^ [15], TcO_4_^−^ [17], Ag^+^, Au^3+^, Cu^2+^ and Hg^2+^ [18], CCl_4_ [16, 22, 23], NO_3_^−^ [14], CrO_4_^2−^ [24–27]) due to their excellent reducing capacity. In these studies, researchers have proposed several mechanisms to explain redox reactions by GRs. [28] For example, Hansen et al. [14] suggested that nitrate (NO_3_^−^) reduction by chloride GR is faster compared to other GRs because chloride interlayer exchange by nitrate, which is then reduced in the interlayer. Similarly, several studies argued that the reduction of chromate (CrO_4_^2−^) occurs in the GR interlayer following anion exchange with chromate [25, 29–31]. In contrast, Thomas et al. [26] proposed that chromate is directly reduced at sulphate GR particle surface sites by electrons shuttled from the particle’s interior via electron hopping [32]. Also, a similar surface process was propsed by Choi et al. [21] and O’Loughlin et al. [15] for the reduction of perchloroethene (C_2_Cl_4_) and U(VI) by GR, respectively. Indeed, C_2_Cl_4_ and U(VI) are unlikely to intercalate into the GR interlayer. Also, the TEM images in O’Loughlin et al. [15] show UO_2_ nanoparticles decorating GR particle edge surfaces after reaction further supporting reduction reaction at GR particle edges [26, 32]. Lastly, recent studies have shown that GR cannot reduce chlorinate ethenes [33], however, if a catalyst such as bone char is added to reaction, chlorinated ethenes can be rapidly reduced by GR, which is contrast to previous study that shows pure GRs cannot reduce chlorinated ethenes [33–35], suggesting that electron transfer can occur in GRs.

These examples demonstrate that despite many experimental studies on GR reductive capacity with contaminants, there is still much confusion about where on GR particles reduction reactions occur, and whether GR hydroxide sheets can shuttle electrons from their interior to the exterior. To unravel the active redox sites on GR hydroxide sheets, we must gain a better understanding of GR electronic properties at a molecular level and evaluate the distribution of Fe(II) and Fe(III) atoms within single GR layers. In this study, we constructed 3 different sized cluster models of a single GR layer and then analyzed its electronic properties using density functional theory (DFT). Specifically, we evaluated the relationship between spin state and total electronic energy and used an implicit solvent model to take into account the interlayer water between the GR hydroxide sheets. The obtained results give new insights into the distribution of Fe(II) and Fe(III) species in GR hydroxide sheets, allowing to make suggestions of GR active redox sites and their role in reactions with contaminants.

## Calculation methods

### Calculation methods

All geometry optimizations were performed with the Turbomole program, v6.5 [36]. The COSMO implicit solvent model [37] with an infinite dielectric constant was combined with Becke–Perdew (BP) functional [38, 39] and triple-ζvalence plus polarization (TZVP) [40] basis set for all calculations. To solve the convergence difficulties of the calculation that result from the high degree of freedom in the models, a higher orbital shifting parameter (0.3) was applied to all calculations.

### GR structure model

**Fig. 1:**
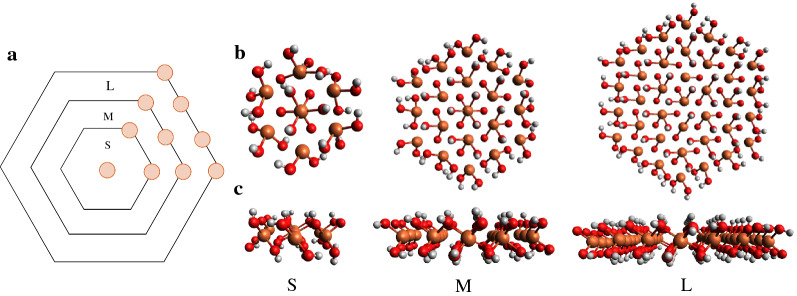
**a** Schematic representation of single layer GR clusters with different sizes: (S) small (GR2 × 6), (M) medium (GR3 × 6) and (L) large (GR4 × 6). The brown dots represent Fe atoms. **b** Top and **c** side view of single layer GR clusters, where Fe atoms are colored brown, O atoms red and H atoms white

The GR structure was based on crystallographic data for sulphate GR (GR_SO4_) provided in Christiansen et al. [3] with following formula: NaFe(II)_6_Fe(III)_3_(SO_4_)_2_(OH)_18_.12H_2_O. GR_SO4_ particles form hexagonal platelets, which consist of hydroxide layers where all octahedral sites are occupied, and the interlayer spaces are filled with octahedrally hydrated sodium and sulphate ions, along with additional water. Here, we only focus on the hydroxide sheet structure (i.e., Fe(II), Fe(III) and OH^−^ ions), for which we constructed three different sized hexagonal clusters representing single GR hydroxide layers, shown in Fig. 1. We modified the edges to ensure that every Fe atom was coordinated to O atoms of six hydroxyl groups. The small (GR2 × 6), medium (GR3 × 6) and large (GR4 × 6) cluster have 2, 3 and 4 Fe atoms, respectively, located on each of the 6 edges (Fig. 1a).

We define the ratio of Fe(II) to Fe(III) atoms in the GR cluster by applying a specific charge to the clusters. This in turn defines the number of unpaired electrons (Fe atoms are assumed to be in high spin states, which means Fe(II) has 4 unpaired electrons and Fe(III) has 5 unpaired electrons; discussed in Sect. 2.3). The tested cluster charges, Fe(II)/Fe(III) ratios and numbers of unpaired electrons are shown for each cluster in Table 1.

**Table 1:** Range of applied Fe(II)/Fe(III) ratios, charges and numbers of unpaired electron (NUE) for GR cluster model

GR cluster model	Fe(II)/Fe(II) ratio	Charge	NUE
GR2 × 6	0/7 to 7/0	− 3 to -10	35 to 28
GR3 × 6	0/19 to 19/0	+ 3 to -16	95 to 76
GR4 × 6	0/37 to 37/0	+ 15 to -22	185 to 148

The details of all tested conditions are given in the Additional file 1: Tables S1–S11

### Multiplicities assumption

**Fig. 2:**
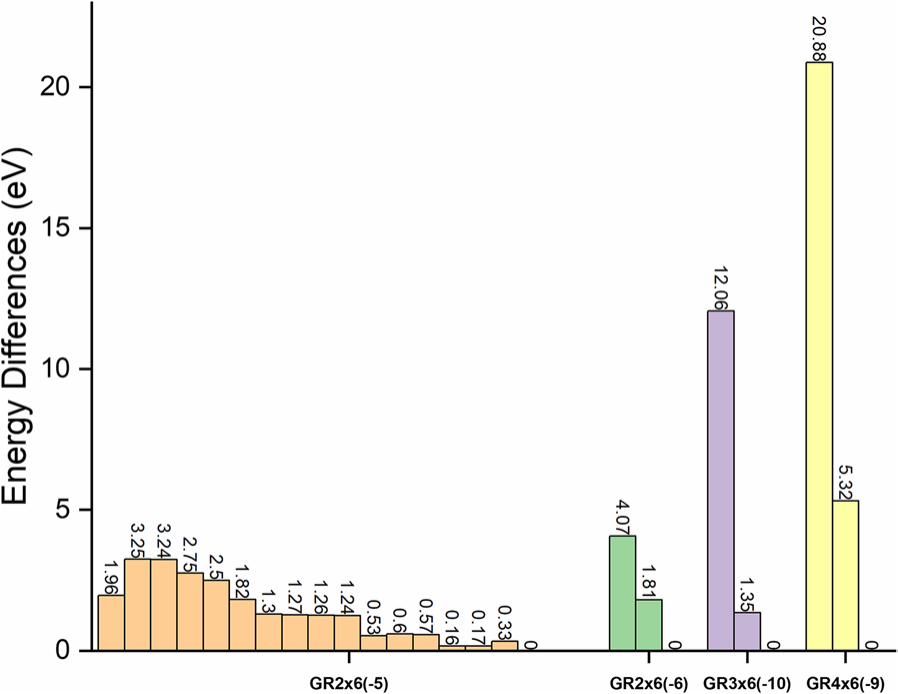
Cluster free energy differences between spin states for the GR clusters. GR2 × 6(− 5): from left to right are spin states with unpaired electrons from 1 to 33 (i.e., 1, 3, 5…). GR2 × 6(− 6), GR3 × 6(− 10) and GR4 × 6(− 9): from left to right are low, intermediate and high spin states respectively. For all models, the energies of the highest spin states are set to be 0

To verify our assumption of Fe electronic occupation, the energy differences between a set of possible spin states for the built GR cluster models were calculated and compared. Specifically, we calculated all possible spin states for GR2 × 6(− 5) (i.e., GR2 × 6 with applied cluster charge of − 5), while for GR2 × 6(− 6), GR3 × 6(− 10), and GR4 × 6(− 9), we only calculated a low, intermediate and high spin state as shown in Fig. 2. Across all performed calculations, the high spin states yield the lowest energy compared to the intermediate and low spin states. Looking at GR2 × 6(− 5), where all possible spin states were calculated, the trends are not perfectly linear between energy and spin state, however, the general trend that the highest spin state have the lowest energy is still evident. These results indicate that for all three GR cluster models, the high spin state is thermodynamically the most favorable state, which is also consistent with Hund’s rule for single atoms.

**Fig. 3:**
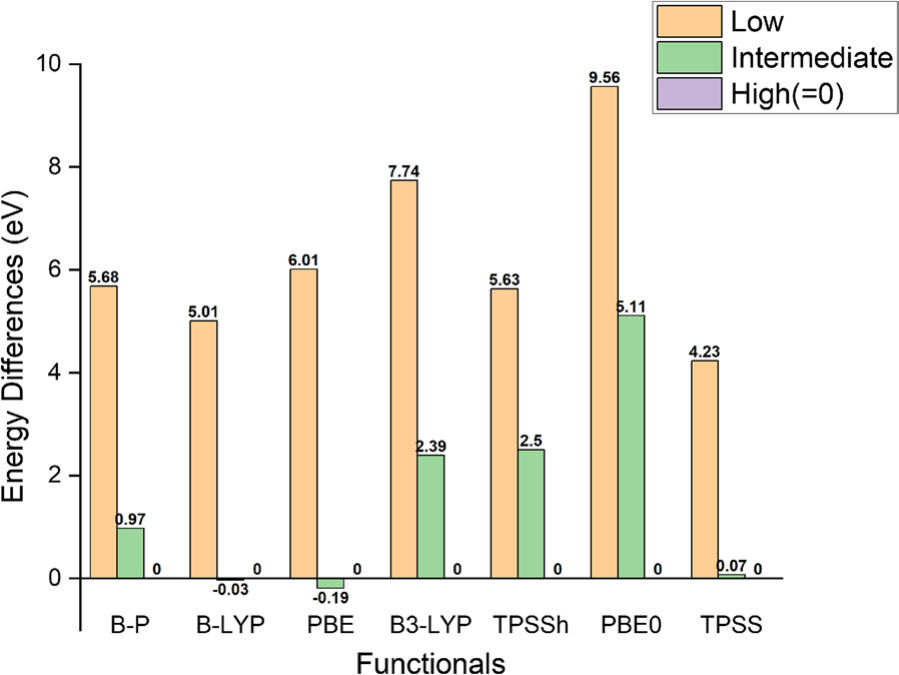
Energy differences between low, intermediate and high spin states of GR2 × 6(− 8) calculated using different DFT functionals

A previous study showed that standard exchange–correlation functionals disfavour high spin state in iron complexes, whereas hybrid and some meta-GGA functionals are generally better at predicting the correct spin state [41]. Despite this, our calculations still show that our models prefer to be in high spin states. However, we still performed a comparison study of different functionals’ preference of spin state for our models. We compared BP86 with 6 other functionals, which represent three basically different approaches of DFT functionals: B-LYP [39, 42] and PBE [43] represent standard exchange–correlation functionals, B3-LYP [42, 44–46], PBE0 [47] and TPSSh [48] represent hybrid functionals, and TPSS [49] represents meta-GGA functional. We used GR2 × 6(− 8) as the example, calculated its fully optimized minimum energy at different spin states using different functionals with same TZVP basis set. Figure 3 shows the energy differences for GR2 × 6(− 8) when iron atoms are at low, intermediate and high spin states, with different functionals. For B-LYP and PBE, the intermediate spin state has the minimum energy, where the energy differences between intermediate and high spin state are very small. All hybrid functionals gave good support for the high spin state, especially PBE0, for which the high spin state has an energy that is 5.11 eV (i.e., 117.84 kcal/mol) lower than the intermediate spin state. B-P only slightly disfavour high spin state compared to hybrid functionals, which is consistent with the previous study [41], however, the energy of high spin state is still significantly lower than the energy of low and intermediate spin state, qualitatively reproducing the spin behaviour of more accurate hybrid functionals. Compared to B-P, TPSS disfavours high spin state considerably more. In our case, the B-P functional show good quality for predicting iron spin state, in qualitative agreement with the more accurate hybrid methods. The use of the BP functional also allows us to apply solvation treatment using COSMO-RS theory. [50]

## Results and discussion

### Electronic properties of the single layer GR3 × 6 model

For the GR3 × 6 cluster, 20 different cluster charge models were calculated. Models with cluster charge from -12 to 0, i.e., Fe(II)/Fe(III) ratios are from 15/4 to 3/16, were obtained by carrying out BP/TZVP optimizations, while models with charges from -13 to -16 yielded distorted structures, and models with charge from + 1 to + 3 exhibited electronic occupations that were not in the ground state. GR3 × 6 with charges from -13 to -16 and from + 1 to + 3 were thus not analyzed here, but are further discussed in the supporting information, Additional file 1: Text S1 and Figure S1. In the following section, we will use the GR3 × 6 model with applied -10 charge, GR3 × 6(− 10), as an example to discuss the structure and electronic property of the single layer GR model. For this cluster, the Fe(II)/Fe(III) ratio is approximately 2.2 which matches the Fe(II)/Fe(III) ratios typically observed for experimentally produced sulphate GR [3].

**Fig. 4:**
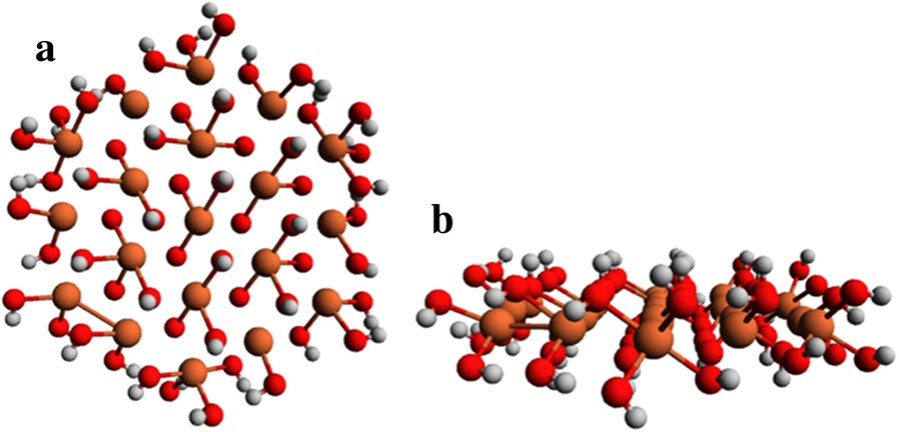
**a** Top and **b** side view of GR3 × 6 with a charge of -10

**Fig. 5:**
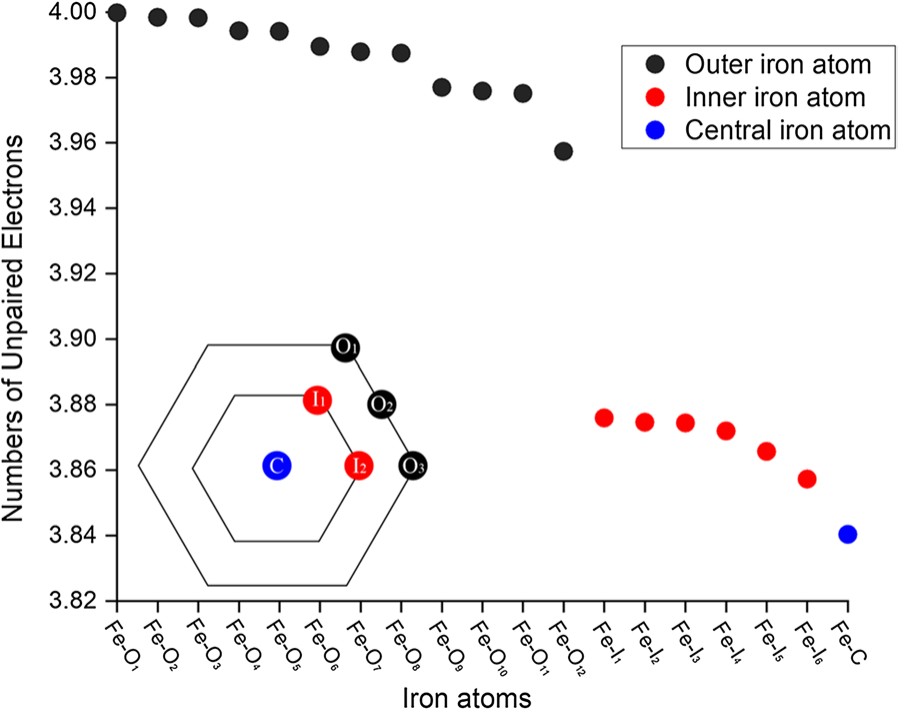
Number of unpaired electrons for Fe atoms in the GR3 × 6(− 10) model structure, ordered according to decreasing number of unpaired electrons. Fe atoms labeled with O, I and C refer to outer, inner and central atoms, respectively

The optimized GR3 × 6(− 10) structure is hexagonal, with all Fe atoms approximately in the same plane (Fig. 4). Given that Fe(III) has more unpaired electrons than Fe(II), we can identify the location of Fe(II) and Fe(III) ions in the structure by assessing the number of unpaired electrons (spin states) in every Fe atom using Mulliken population analysis. The results of this analysis for GR3 × 6(− 10) (Fig. 5) show that Fe atoms at the edges of the simulated GR layer have a higher number of unpaired electrons (i.e., higher spin states) compare to Fe atoms in the interior of the GR layer, indicated by a gap of 0.1. Additionally, the central Fe atom seems to have the lowest number of unpaired electron (i.e., lowest spin state). Thus, the reducing capacity of outer Fe atoms is lower compared to inner Fe atoms, with the central Fe atom having the highest reducing capacity. This means that the reductive capacity across the GR sheet structure is quite uneven, with the inner Fe atoms having Fe(II) like character, while the outer Fe atoms are more Fe(III) like.

**Fig. 6:**
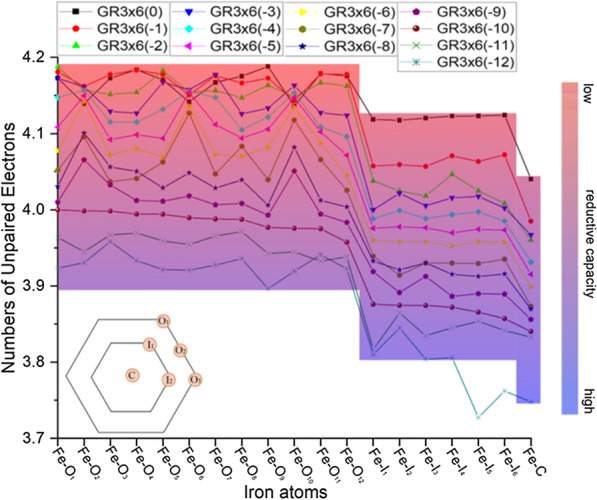
Numbers of unpaired electrons for Fe irons atoms in all obtained GR3 × 6 models. Fe atoms labeled with O, I and C refers to outer, inner and central atoms, respectively

To verify the observations made for GR3 × 6(− 10), the distribution of unpaired electrons was also calculated for all other stable GR3 × 6 models (i.e., with charge from -12 to 0 i.e., Fe(II)/Fe(III) ratio from 15/4 to 3/16) shown in Fig. 6. Identical to the results of GR3 × 6(− 10), the number of unpaired electrons is considerably higher (i.e., spin states are higher) for outer Fe atoms compared to inner Fe atoms in all 13 model structures. Note that the clear drop in spin state between outer and inner Fe atoms is also still visible when averaging the number of unpaired electrons across all outer and inner Fe atoms, respectively, or by comparing minimum and maximum values for outer and inner Fe atoms, respectively (Table 2). Also, in most models (except for GR3 × 6(− 11) and GR3 × 6(− 12)), the central Fe atom still exhibits the lowest spin state as initially observed for GR3 × 6(− 10) (Fig. 6, Table 2). Overall, all 13 GR3 × 6 models reaffirmed the uneven distribution of reducing capacity (i.e., Fe(II) and Fe(III) atoms) across the single GR sheet. We note that this trend was consistent across the wide range of cluster charge (Fe(II)/Fe(III) ratio) we investigated.

**Table 2:** Relative differences in the number of unpaired electrons (NUE) between outer, inner and central Fe atoms for the simulated GR3 × 6 models

	0	− 1	− 2	− 3	− 4	− 5	− 6	− 7	− 8	− 9	− 10	− 11	− 12	Ave
Δ1_O-I_	0.012	0.071	0.101	0.102	0.097	0.094	0.085	0.087	0.071	0.065	0.081	0.068	0.051	0.076
Δ2_O-I_	0.046	0.107	0.134	0.137	0.137	0.136	0.132	0.136	0.119	0.117	0.116	0.112	0.135	0.132
Δ1_I-C_	0.077	0.072	0.048	0.033	0.054	0.055	0.054	0.041	0.043	0.030	0.017	*-0.016*	*-0.020*	0.048
Δ2_I-C_	0.081	0.078	0.067	0.043	0.061	0.059	0.059	0.056	0.052	0.042	0.030	0.010	0.044	0.052

The cluster charges are differing from 0 to − 12 (GR3 × 6(0) to GR3 × 6(− 12)), i.e. Fe(II)/Fe(III) ratios from 3/16 to 15/4. Δ1_O-I_ denotes the difference between minimum and maximum NUE of outer and inner Fe, respectively; Δ2_O-I_ denotes the difference between average NUEs of outer and inner Fe atoms, respectively; Δ1_I-C_ denotes the difference between minimum and maximum NUE of inner and central Fe atoms, respectively; Δ2_I-C_ denotes the difference between average NUEs of inner and central Fe atoms, respectively. The NUE details of GR3×6 models can be seen in the Additional file 1: Table S3

### Cluster size effects

**Fig. 7:**
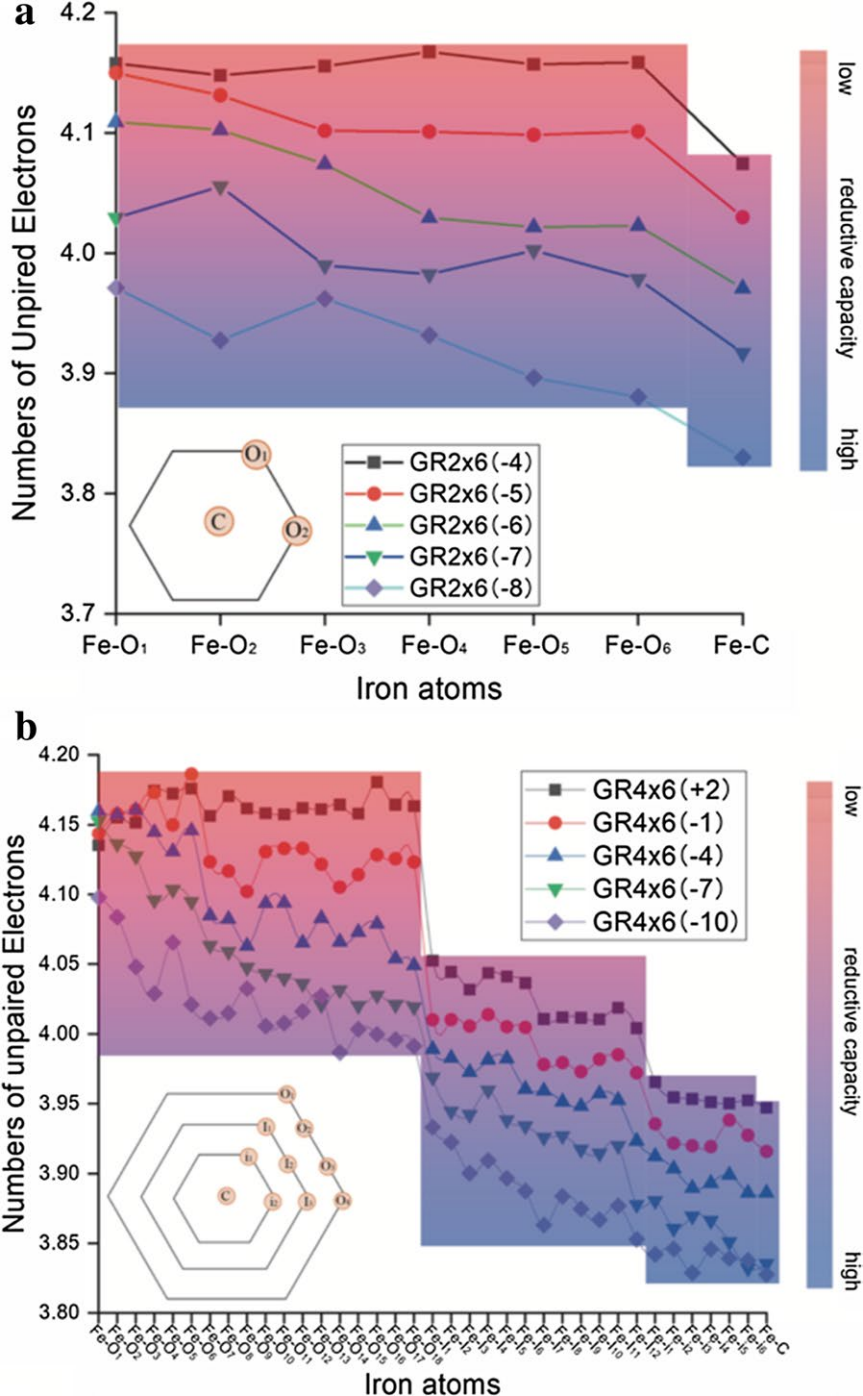
Number of unpaired electrons for the Fe atoms in the stable **a** GR2 × 6 models (all 5 shown) and **b** GR4 × 6 models (only 5 shown for clarity, all 23 models are shown in Additional file 1: Figure S2). Fe atoms labeled with O, I and C refer to outer, inner and central atoms, respectively

To ensure results observed for the GR3 × 6 models are not dependent on cluster size, we performed similar electronic property analyses for a smaller (GR2 × 6) and a larger (GR4 × 6) GR single layer structure, shown in Fig. 7a, b. For those two cluster sizes, 8 and 38 Fe(II)/Fe(III) ratios were examined, and 5 and 23 yielded thermodynamically stable structures after geometry optimization, respectively.

The spin state distributions calculated for the GR2 × 6 and GR4 × 6 models showcase the same drop in the number of unpaired electrons between outer and central iron atoms as shown for the GR3 × 6 model. This is also verified by comparison of minimum and maximum spin values, or average values (Tables S2 and S4 in Additional file 1) has done for the GR3 × 6 model. Thus all three different sized models support the same electronic configuration for GR hydroxide sheets, with Fe(II) like atoms positioned in the middle of the GR sheet and Fe(III) like atoms on GR sheet edges. The consistency in our observations between different investigated Fe(II)/Fe(III) ratios and cluster sizes strongly suggest that our models provide a reasonable description of the electronic properties of a single GR hydroxide sheet.

### Spin contamination

In open-shell system, unrestricted calculations have different set of orbitals for alpha electrons and beta electrons. In this case, spin contamination can occur because the expected value of total spin angular momentum operator, S^2^, is no longer commensurate with expected spin state of the system [51]. A large spin contamination indicates a failure of the applied computational method. However, because S^2^ is a two-electron operator, its meaning in density functional theory is diffuse. A previous study argued that S^2^ can be assigned a diagnostic value also within DFT [52, 53], and we would like to provide the details of spin contamination in our green rust models.

The spin contamination for the three different sized GR models was determined as the difference between the calculated S^2^ and the ideal value of S(S + 1), i.e., ΔS^2^ = S^2^-S(S-1). Calculated ΔS^2^ values (Table 3) are very small, especially considering the high spin states in the GR models calculated here. It is worth mentioning that the spin contamination increases as the GR model size is increased, but it decreases with as increase in multiplicities within the same sized GR model. Nevertheless, the highest ΔS^2^ value of 0.25 obtained for GR4 × 6(− 16) is still negligible considering its large number of unpaired electrons.

**Table 3:** ΔS^2^ values for all stable GR2 × 6, GR3 × 6 and GR4 × 6 models calculated using B-P functional

Model	ΔS^2^	Model	ΔS^2^	Model	ΔS^2^	Model	ΔS^2^	Model	ΔS^2^	Model	ΔS^2^
GR2 × 6(− 4)	0.03	GR3 × 6(− 2)	0.09	GR3 × 6(− 9)	0.10	GR4 × 6(+ 3)	0.18	GR4 × 6(− 4)	0.20	GR4 × 6(− 11)	0.22
GR2 × 6(− 5)	0.03	GR3 × 6(− 3)	0.09	GR3 × 6(− 10)	0.11	GR4 × 6(+ 2)	0.18	GR4 × 6(− 5)	0.20	GR4 × 6(− 12)	0.23
GR2 × 6(− 6)	0.03	GR3 × 6(− 4)	0.09	GR3 × 6(− 11)	0.11	GR4 × 6(+ 1)	0.18	GR4 × 6(− 6)	0.20	GR4 × 6(− 13)	0.22
GR2 × 6(− 7)	0.04	GR3 × 6(− 5)	0.10	GR3 × 6(− 12)	0.13	GR4 × 6(0)	0.18	GR4 × 6(− 7)	0.21	GR4 × 6(− 14)	0.23
GR2 × 6(− 8)	0.05	GR3 × 6(− 6)	0.10	GR4 × 6(+ 6)	0.17	GR4 × 6(− 1)	0.19	GR4 × 6(− 8)	0.21	GR4 × 6(− 15)	0.23
GR3 × 6(0)	0.08	GR3 × 6(− 7)	0.10	GR4 × 6(+ 5)	0.17	GR4 × 6(− 2)	0.19	GR4 × 6(− 9)	0.21	GR4 × 6(− 16)	0.25
GR3 × 6(− 1)	0.08	GR3 × 6(− 8)	0.10	GR4 × 6(+ 4)	0.17	GR4 × 6(− 3)	0.19	GR4 × 6(− 10)	0.21		

We also evaluated the effect of different DFT functionals on spin contamination using the GR2 × 6(− 8) model as an example (Table 4), second order Møller-Plesset (MP2) perturbation theory [54–57] also applied to determine the spin contamination. All methods show same result of a low degree of spin contamination, with the B-P functional only slightly higher than the hybrid functionals.

**Table 4:** ΔS^2^ values of GR2 × 6(− 8) with different DFT functionals and MP2 method. All the results were obtained with fully optimized geometry for the corresponding method

	B-P	B-LYP	PBE	B3-LYP	TPSSh	PBE0	TPSS	MP2
ΔS^2^(GR2 × 6(− 8))	0.047	0.042	0.051	0.038	0.039	0.038	0.040	0.049

### Mulliken and natural bond orbital analysis

**Fig. 8:**
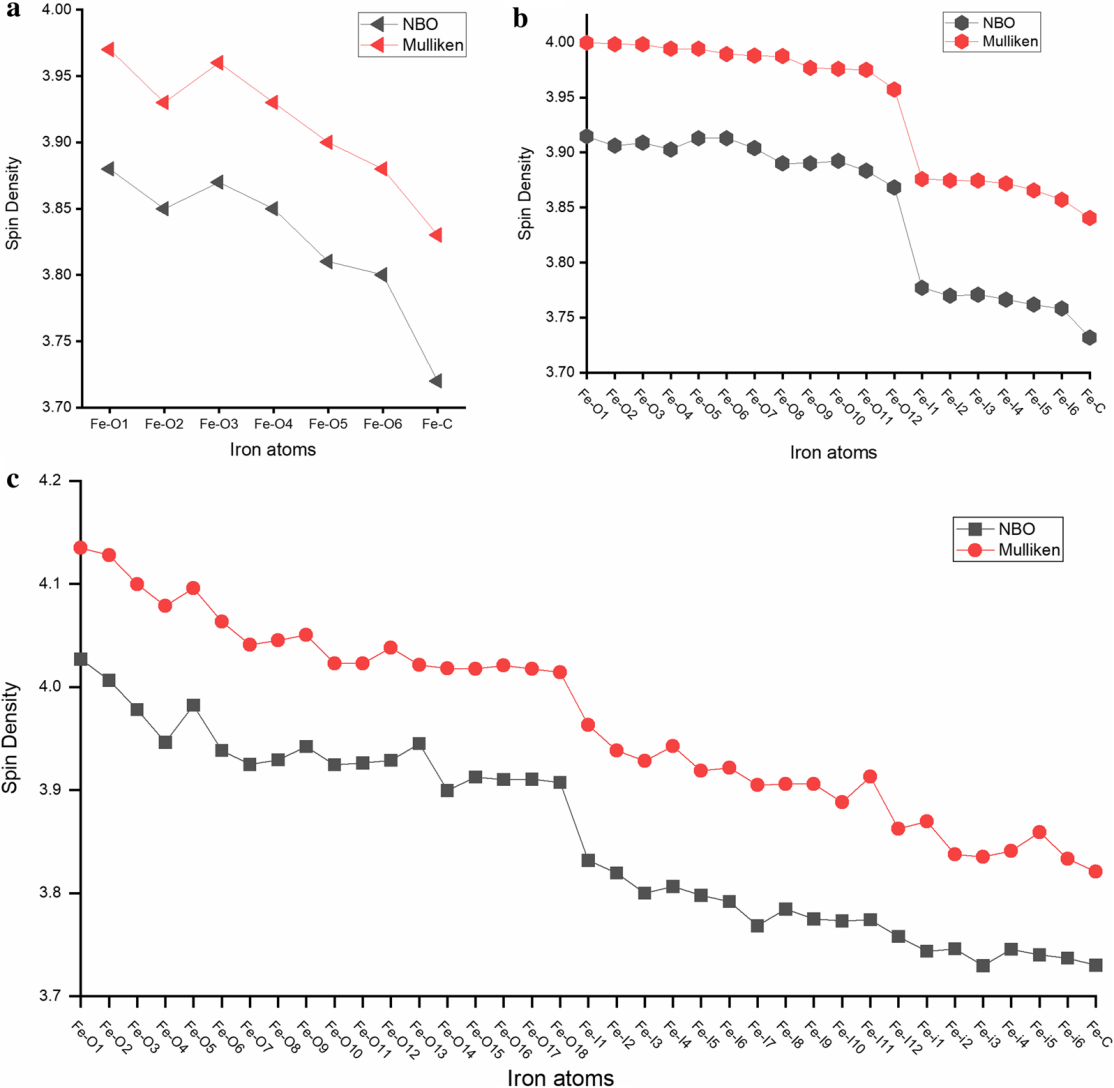
The comparison of spin density of Fe atoms obtained by Mulliken and Natural Bond Orbital (NBO) population analysis for **a** GR2 × 6(− 8), **b** GR3 × 6(− 10) and **c** GR4 × 6(− 10)

Mulliken method is known as the oldest and cheapest way to obtain spin density and atomic charges, but it is also criticised for its lacking polarization effects and basis set dependence [58, 59]. Natural bond orbital (NBO) analysis is considered to be more reliable because it takes electron density and polarization effects into account. Hence, we evaluated the suitability of both the Mulliken and the NBO population analysis. The Mulliken unpaired electrons and NBO spin density for Fe atoms in the three GR models, GR2 × 6(− 8), GR3 × 6(− 10) and GR4 × 6(− 10), is shown in Fig. 8 (at B-P/TZVP level). Although absolute values between the Mulliken and NBO spin density differ, the spin density distributions among iron atoms in our models are basically identical.

### Functional effects

The spin density distributions were calculated for GR2 × 6(− 8) and GR3 × 6(− 8) using the different DFT functionals discussed earlier (“Multiplicities assumption” section) and they are shown in the Additional file 1: Figures S3 (GR2 × 6(− 8) with different functionals), S4 (GR2 × 6(− 8) with MP2) and S5 (GR3 × 6(− 10) with PBE0). Two types of spin density distributions are observed: (1) The pure functionals, BLYP, PBE and TPSS, produced similar distributions as the B-P functional in that the center Fe atoms show the lowest spin density, while the outer Fe atoms show relatively high spin states; (2) The hybrid functionals, B3LYP, TPSSh, PBE0 and MP2, include exact exchange, and they show only two discreet values (except for TPSSh) as opposed to the smeared out electronic distribution of pure DFT functionals. These two values can be identified as Fe(III) and Fe(II). All high spin Fe(III) are located on GR sheet edges, meaning that all inner Fe atoms are Fe(II).

Although the pure DFT functionals do not localize the electrons correctly, the distribution trends agree with trends observed for hybrid functionals, which localize the electrons accurately into Fe(II) and Fe(III). Additionally in Additional file 1: Figures S3 and S4, the two Fe atoms that are predicted to be Fe(III) by the hybrid functionals are the two atoms with the highest calculated spin in the pure DFT calculations. Thus, all functionals investigated (pure and hybrid) reveal the same tendency that the outer Fe atoms on average have higher spin state compared to inner Fe atoms. The calculated spin density of the larger cluster GR3 × 6(− 10) with PBE0 also shows a consistent performance, with all six Fe(III) located on the edge of the cluster. If the Fe(III) were randomly distributed, there would only be a 3% likelihood that all Fe(III) would happen to be located at the edge sites, strengthening our conclusions about Fe(III) preferring to be locate at the edge sites.

## Conclusions and implications

In this work, three different sized cluster models of single GR hydroxide layers were built to study their electronic properties using density functional theory. The calculations showed that a high spin state is favored thermodynamically for the single layer GR model and that a minimum amount of Fe(III) are required, i.e., Fe(II)/Fe(III) ratios between 0.2 and 5, to maintain the hexagonal shape of the GR structure. These ratios are in agreement with ratios measured of synthetic and natural GR samples that typically range between 2 and 3 [3]. All three cluster models showed that the spin states of edge Fe atoms are significantly higher compared to Fe atoms located in the GR interior. This in turn means that Fe(II) and Fe(III) ions are unevenly distributed across the GR hydroxide sheet structure, with edge Fe atoms being more Fe(III)-like, while inner Fe atoms are more Fe(II)-like; thus the GR interior is more reducing compared to the edge. The trends we found were consistent among several pure DFT calculations, hybrid functionals as well as MP2. A reason that may explain why we observed this uneven Fe(II)/Fe(III) distribution is that the chemical environment between edge and inner Fe atoms is different. The edge atoms have larger degree of flexibility compared to the more constrained inner atoms, which are more or less fixed in crystallographic positions. This could allow for a more unconstrained relaxation, including the electronic degrees of freedom. Furthermore, the edge iron atoms bind to three or four hydroxyl ions, instead of bridging oxygen between iron atoms as done by the inner iron atoms. The different chemical environment creates a nonisotropic iron site, which could affect the electronic properties as well, including via a local dipole effect.

Evidently, this GR model excluds the presence of charge-balancing interlayer anions, which potentially could affect the local Fe(II)/Fe(III) distribution, i.e., electronic properties, across the GR hydroxide sheet. However, it is still worth making some initial comparisons to experimental observations here. Taking the example of the reduction of chromate, Cr(VI), by sulphate GR, some studies argued that Cr(VI) is reduced in the interlayer after sulphate exchange with chromate [25, 29–31], which would support our observations. In contrast, others argued for Cr(VI) reduction to occur at GR_SO4_ particle edges [26]. Similarly, the reductions of U(VI) [15] and C_2_Cl_4_ [21] by GR are argued to occur on the GR edges which seems to be contrary to our finding that the reduction capacity tends to be higher in the interior of GR crystals. However, the charge hopping mechanism proposed by Wander et al. [32] could explain why reduction can occur on the edge, even if it is less reactive than the central part of GR. Indeed, electron microscopy images of Cr(VI) reacted sulphate GR often show reaction rims with oxidized Fe (oxyhydr) oxide phases, while the GR interiors seem to get fully dissolved [25], which may be another indication that GR interiors are more reactive, i.e., more reducing, and that electrons can readily transfer from GR interior to its edges.

Lastly, our study also suggests that the B-P functional with a TZVP basis set gives reasonable results for the electronic distribution in GR, which opens up possibilities for modelling solvation behavior of interlayer molecules using the implicit solvent method COSMO-RS [50].

## Supplementary Information

The full list of Fe(II)/Fe(III) ratios, the number of unpaired electrons in every Fe atoms of GR2 × 6, GR3 × 6 and GR4 × 6, and discussion of Fe(II)/Fe(III) ratio and structure stability are shown in additional file. The discussion about the effect of dielectric constant on the Fe spins (Additional file 1: Text S2, Tables S5–S7 and Figure S6), and magnetic property analysis (Additional file 1: Text S3 and Tables S8–S11) are can also be seen in Additional file 1.

## Supplementary Information

**Additional file 1.** Additional tables and figures.

## Abbreviations

GR: Green rust
GRSO4: Sulphate green rust
LDH: Layered double hydroxide
NUE: Number of unpaired electrons
DFT: Density Functional Theory
COSMO: Conductor like screening model,
NBO: Natural bond orbital
