# Supplementary material for: A density functional theory study of Fe(II)/Fe(III) distribution in single layer green rust: a cluster approach
Source: Geochem Trans. 2021 Jun 11;22:3. doi: 10.1186/s12932-021-00076-0 (PMC8194116; doi:10.1186/s12932-021-00076-0)
Supplement: Supplementary file 1 — Additional file 1. Additional tables and figures. [file 12932_2021_76_MOESM1_ESM.docx]

Supporting Information

A Density Functional Theory Study of Fe(II)/Fe(III) Distribution in Single Layer Green Rust: A Cluster Approach

Weichao Sun^*a^, Dominique J. Tobler^a,b^, Martin P. Andersson^c^

^a^Nano-Science Center, Department of Chemistry, University of Copenhagen, 2100 Copenhagen, Denmark

^b^Department of Plant and Environmental Sciences, University of Copenhagen, Thorvaldsensvej 40, 1871 Frederiksberg C, Denmark

^c^Department of Chemical and Biochemical Engineering, Technical University of Denmark, 2800 Kgs. Lyngby, Denmark

[^*^wsun@chem.ku.dk](mailto:*wsun@chem.ku.dk)

# Table S1. The Fe(II)/Fe(III) ratios, charges and numbers of unpaired electron for all GR clusters.

|  | GR2x6 | |  |  | GR3x6 |  |  | GR4x6 |  |
| --- | --- | --- | --- | --- | --- | --- | --- | --- | --- |
| Fe^II^/Fe^III^ ratio | charge | NUE | | Fe^II^/Fe^III^ ratio | charge | NUE | Fe^II^/Fe^III^ ratio | charge | NUE |
| 0/7 | -3 | 35 | | 0/19 | +3 | 95 | 0/37 | +15 | 185 |
| 1/6^*^ | -4 | 34 | | 1/18 | +2 | 94 | 1/36 | +14 | 184 |
| 2/5 | -5 | 33 | | 2/17 | +1 | 93 | 2/35 | +13 | 183 |
| 3/4 | -6 | 32 | | 3/16 | 0 | 92 | 3/34 | +12 | 182 |
| 4/3 | -7 | 31 | | 4/15 | -1 | 91 | 4/33 | +11 | 181 |
| 5/2 | -8 | 30 | | 5/14 | -2 | 90 | 5/32 | +10 | 180 |
| 6/1 | -9 | 29 | | 6/13 | -3 | 89 | 6/31 | +9 | 179 |
| 7/0 | -10 | 28 | | 7/12 | -4 | 88 | 7/30 | +8 | 178 |
|  |  |  | | 8/11 | -5 | 87 | 8/29 | +7 | 177 |
|  |  |  | | 9/10 | -6 | 86 | 9/28 | +6 | 176 |
|  |  |  | | 10/9 | -7 | 85 | 10/27 | +5 | 175 |
|  |  |  | | 11/8 | -8 | 84 | 11/26 | +4 | 174 |
|  |  |  | | 12/7 | -9 | 83 | 12/25 | +3 | 173 |
|  |  |  | | 13/6 | -10 | 82 | 13/24 | +2 | 172 |
|  |  |  | | 14/5 | -11 | 81 | 14/23 | +1 | 171 |
|  |  |  | | 15/4 | -12 | 80 | 15/22 | 0 | 170 |
|  |  |  | | 16/3 | -13 | 79 | 16/21 | -1 | 169 |
|  |  |  | | 17/2 | -14 | 78 | 17/20 | -2 | 168 |
|  |  |  | | 18/1 | -15 | 77 | 18/19 | -3 | 167 |
|  |  |  | | 19/0 | -16 | 76 | 19/18 | -4 | 166 |
|  |  |  | |  |  |  | 20/17 | -5 | 165 |
|  |  |  | |  |  |  | 21/16 | -6 | 164 |
|  |  |  | |  |  |  | 22/15 | -7 | 163 |
|  |  |  | |  |  |  | 23/14 | -8 | 162 |
|  |  |  | |  |  |  | 24/13 | -9 | 161 |
|  |  |  | |  |  |  | 25/12 | -10 | 160 |
|  |  |  | |  |  |  | 26/11 | -11 | 159 |
|  |  |  | |  |  |  | 27/10 | -12 | 158 |
|  |  |  | |  |  |  | 28/9 | -13 | 157 |
|  |  |  | |  |  |  | 29/8 | -14 | 156 |
|  |  |  | |  |  |  | 30/7 | -15 | 155 |
|  |  |  | |  |  |  | 31/6 | -16 | 154 |
|  |  |  | |  |  |  | 32/5 | -17 | 153 |
|  |  |  | |  |  |  | 33/4 | -18 | 152 |
|  |  |  | |  |  |  | 34/3 | -19 | 151 |
|  |  |  | |  |  |  | 35/2 | -20 | 150 |
|  |  |  | |  |  |  | 36/1 | -21 | 149 |
|  |  |  | |  |  |  | 37/0 | -22 | 148 |

# Text S1. The structure and Fe(II)/Fe(III) ratio

We calculated all the possible Fe(II)/Fe(III) ratio for our GR models. The structure analysis shows that to maintain the hexagonal shape, a certain amount of Fe(II) is needed. This is exemplified in Figure S1 for GR2x6 with minus 9 charge (i.e., GR2x6(-9)), GR3x6 with -13 charge and GR4x6 with -17 charge, having respective Fe(II)/Fe(III) ratios of 6, 5.3 and 6.4. These model structures have insufficient Fe(III) atoms leading to distorted structures, as evident by hydroxyls (OH^-^) leaving the GR sheets (black arrows in Figure S1) and the fact that Fe atoms are not in the same plane. In our calculation, distortions appear at Fe(II)/Fe(III) ratio > 2.5 in the small (GR2x6), > 3.75 in the medium (GR3x6), >5.1 in the large (GR4x6) GR cluster. To ensure the GR hexagonal structure is maintained in our models, Fe(II)/Fe(III) ratio > 5 were excluded in further evaluations. It is also worth noting that if the Fe(II)/Fe(III) ratio is too small, no stable electronic occupation could be obtained, this situation occurs at Fe(II)/Fe(III) < 0.17 in small (GR2x6), < 0.19 in medium (GR3x6), <0.32 in large (GR4x6) GR clusters.

# Figure S1. The structures of a) GR2x6(-9), b) GR3x6(-13), c) GR4x6(-17).


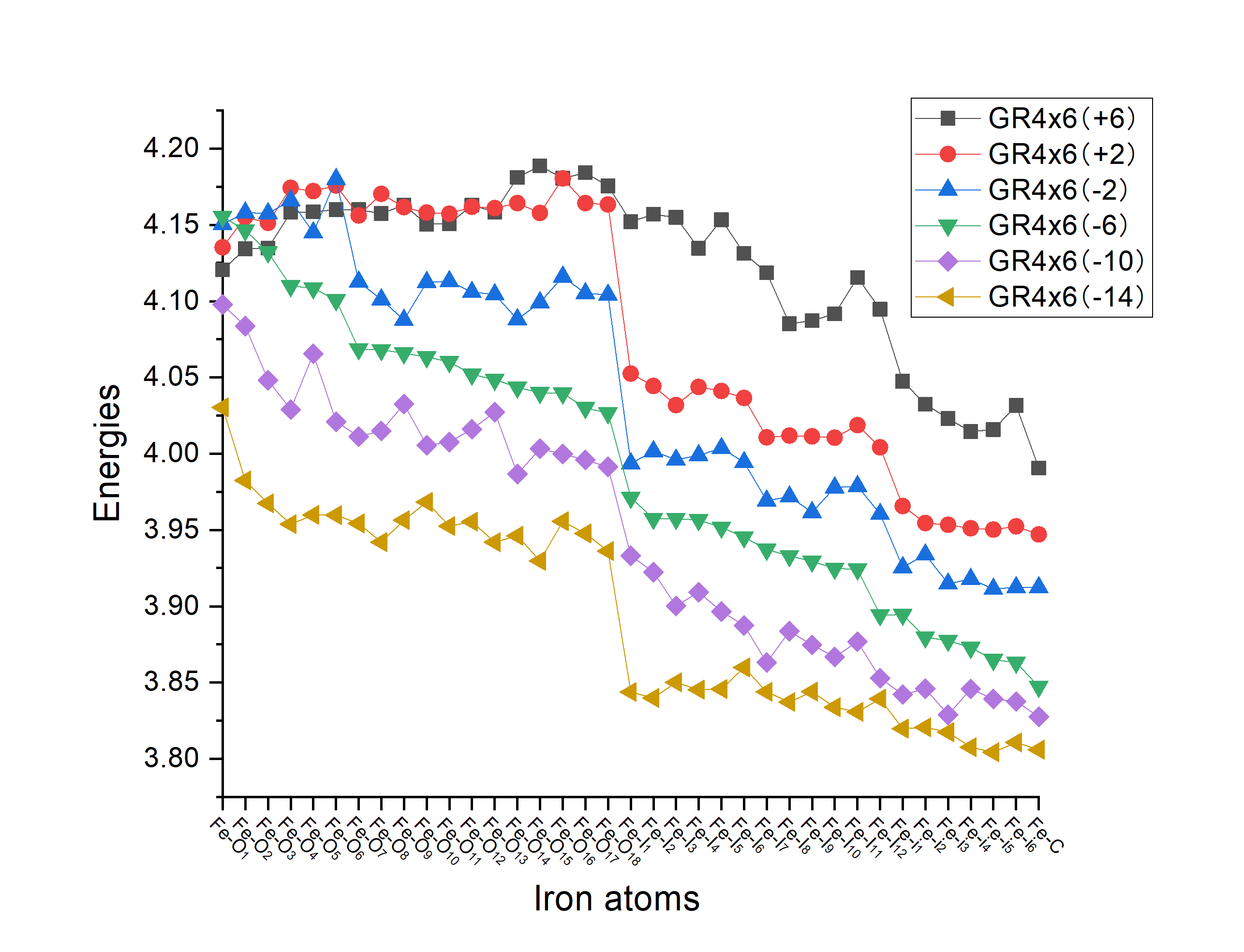


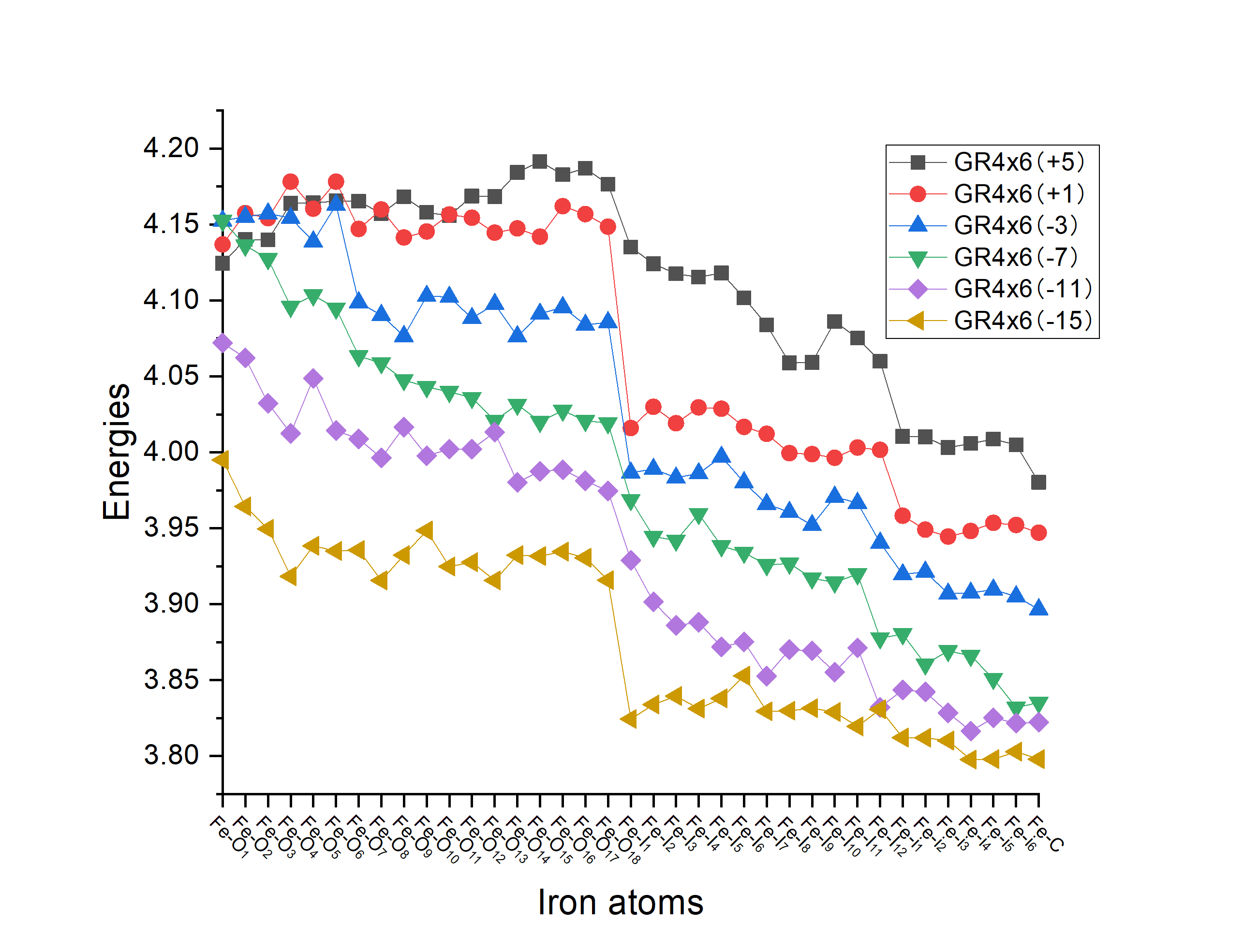


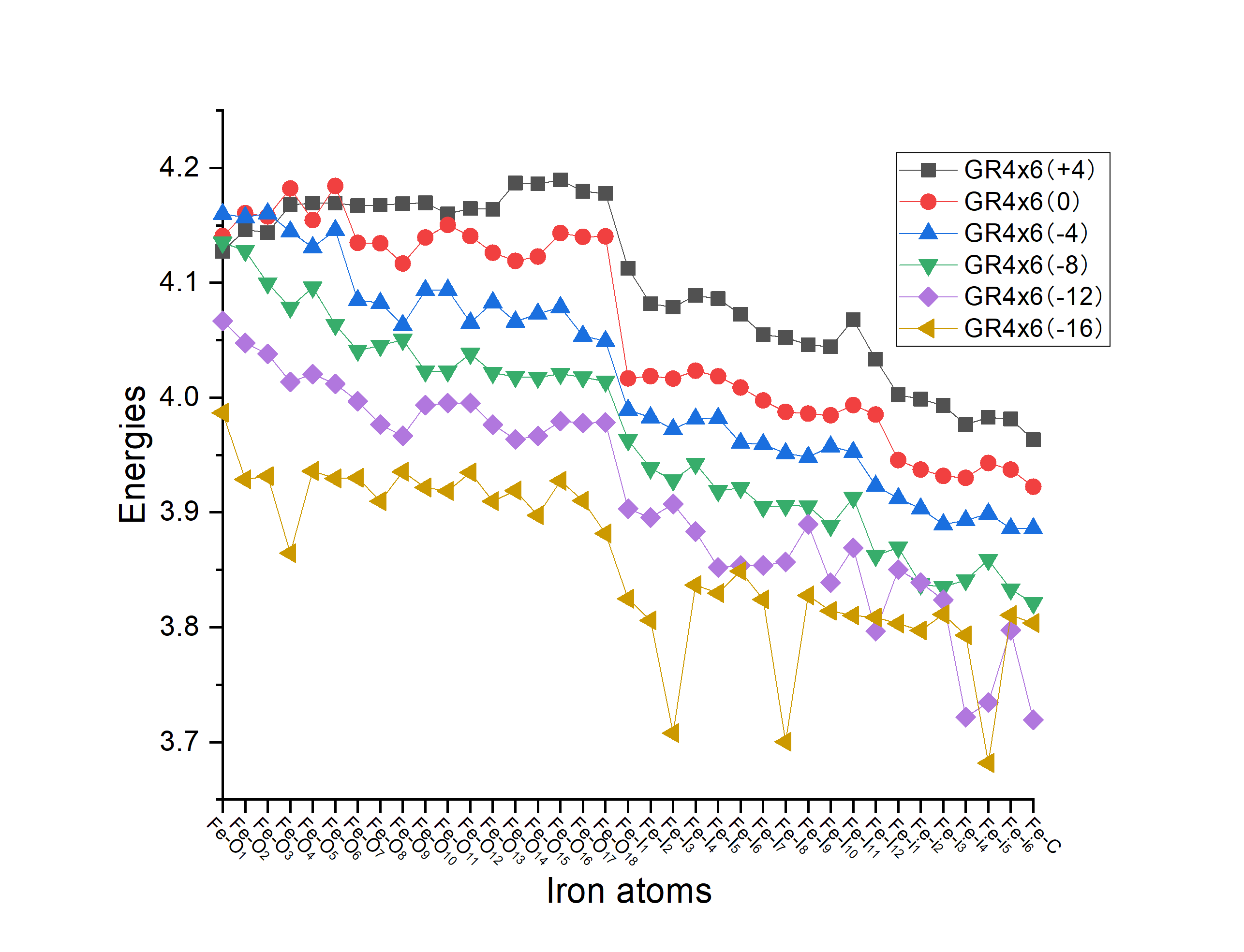


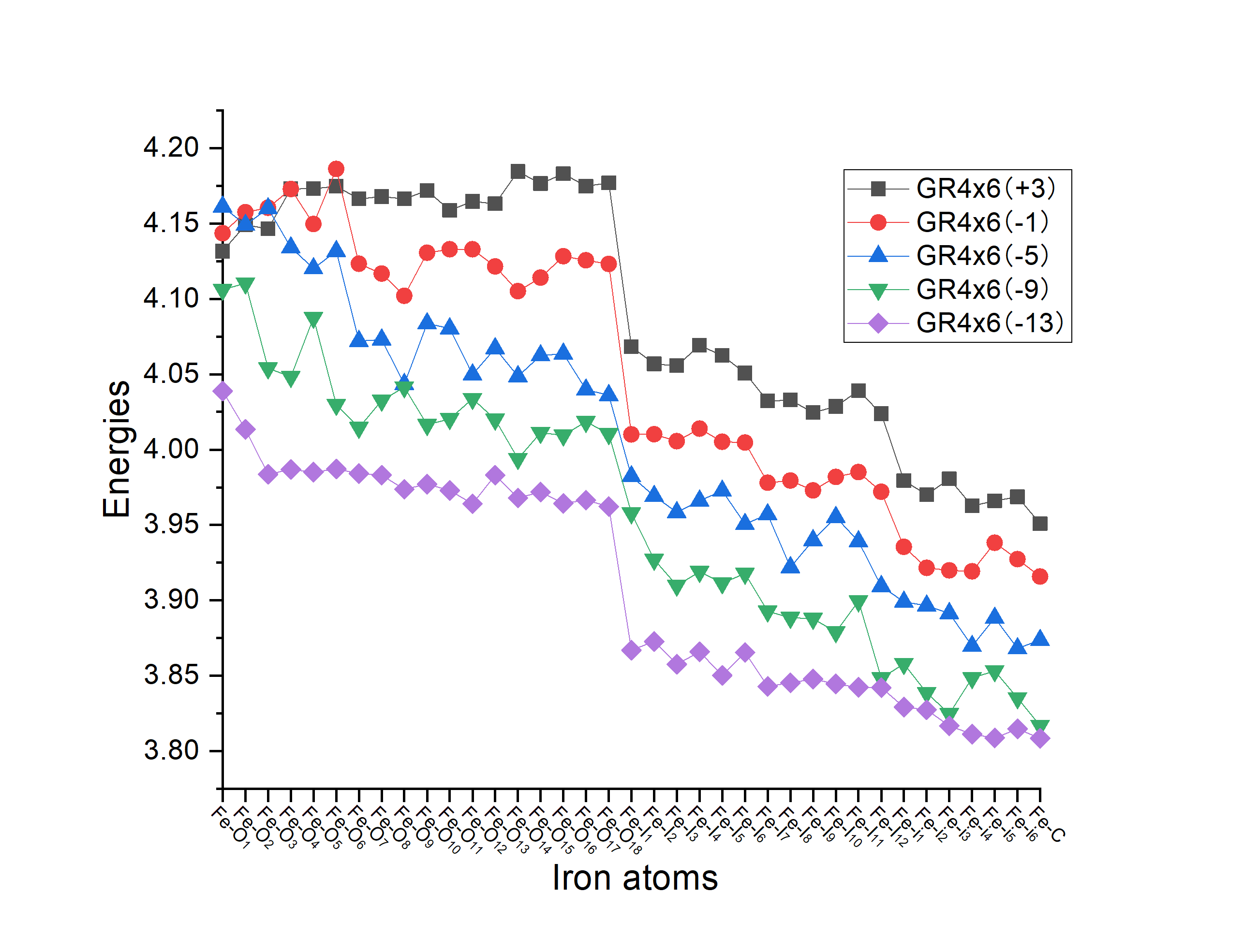


# Figure S2. Numbers of unpaired electrons in Fe atoms in all GR4x6 models.

Table S2. Numbers of unpaired electrons (NUE) in Fe atoms of GR2x6 models, and relative differences in the NUE between outer and central Fe atoms for the simulated GR2x6 models with cluster charge differing from -4 to -8. Δ1_O-C_ denotes the difference between minimum and maximum NUE of outer and central Fe atoms, respectively; Δ2_O-C_ denotes the difference between average NUEs of outer and central Fe atoms, respectively.

|  | -4 | -5 | -6 | -7 | -8 |
| --- | --- | --- | --- | --- | --- |
| Fe-O_1_ | 4.156 | 4.102 | 4.074 | 3.990 | 3.962 |
| Fe-O_2_ | 4.159 | 4.101 | 4.023 | 3.978 | 3.880 |
| Fe-O_3_ | 4.148 | 4.131 | 4.102 | 4.055 | 3.927 |
| Fe-O_4_ | 4.167 | 4.101 | 4.029 | 3.982 | 3.932 |
| Fe-O_5_ | 4.158 | 4.150 | 4.109 | 4.029 | 3.971 |
| Fe-O_6_ | 4.157 | 4.098 | 4.022 | 4.002 | 3.896 |
| Fe-C | 4.075 | 4.030 | 3.971 | 3.917 | 3.830 |
| Δ1_O-C_ | 0.073 | 0.068 | 0.051 | 0.062 | 0.050 |
| Δ2_O-C_ | 0.081 | 0.086 | 0.086 | 0.093 | 0.091 |

# Table S3. Number of unpaired electrons (NUE) in Fe atoms of GR3x6 models.

|  | 0 | -1 | -2 | -3 | -4 | -5 | -6 | -7 | -8 | -9 | -10 | -11 | -12 |
| --- | --- | --- | --- | --- | --- | --- | --- | --- | --- | --- | --- | --- | --- |
| Fe-O_1_ | 4.179 | 4.178 | 4.167 | 4.127 | 4.108 | 4.102 | 4.087 | 4.066 | 4.012 | 3.994 | 3.975 | 3.932 | 3.941 |
| Fe-O_2_ | 4.136 | 4.143 | 4.148 | 4.163 | 4.153 | 4.147 | 4.135 | 4.118 | 4.082 | 4.051 | 3.976 | 3.945 | 3.919 |
| Fe-O_3_ | 4.184 | 4.183 | 4.154 | 4.127 | 4.115 | 4.098 | 4.080 | 4.041 | 4.050 | 4.012 | 3.994 | 3.970 | 3.933 |
| Fe-O_4_ | 4.167 | 4.176 | 4.156 | 4.177 | 4.147 | 4.112 | 4.072 | 4.047 | 4.028 | 4.006 | 3.988 | 3.966 | 3.927 |
| Fe-O_5_ | 4.175 | 4.166 | 4.147 | 4.126 | 4.105 | 4.094 | 4.070 | 4.083 | 4.039 | 4.008 | 3.987 | 3.971 | 3.936 |
| Fe-O_6_ | 4.139 | 4.162 | 4.157 | 4.161 | 4.157 | 4.150 | 4.142 | 4.095 | 4.101 | 4.065 | 3.998 | 3.945 | 3.931 |
| Fe-O_7_ | 4.188 | 4.172 | 4.163 | 4.133 | 4.122 | 4.106 | 4.082 | 4.040 | 4.006 | 3.993 | 3.977 | 3.943 | 3.896 |
| Fe-O_8_ | 4.170 | 4.178 | 4.182 | 4.167 | 4.132 | 4.094 | 4.070 | 4.063 | 4.029 | 4.011 | 3.994 | 3.959 | 3.922 |
| Fe-O_9_ | 4.176 | 4.177 | 4.162 | 4.124 | 4.096 | 4.071 | 4.045 | 4.026 | 4.004 | 3.983 | 3.957 | 3.938 | 3.923 |
| Fe-O_10_ | 4.142 | 4.151 | 4.154 | 4.157 | 4.156 | 4.154 | 4.136 | 4.127 | 4.048 | 4.018 | 3.989 | 3.954 | 3.921 |
| Fe-O_11_ | 4.173 | 4.177 | 4.151 | 4.129 | 4.115 | 4.092 | 4.072 | 4.037 | 4.056 | 4.033 | 3.998 | 3.967 | 3.958 |
| Fe-O_12_ | 4.173 | 4.181 | 4.187 | 4.172 | 4.147 | 4.108 | 4.077 | 4.052 | 4.030 | 4.010 | 4.000 | 3.964 | 3.923 |
| Fe-I_1_ | 4.120 | 4.057 | 4.018 | 4.005 | 3.988 | 3.976 | 3.958 | 3.930 | 3.930 | 3.913 | 3.874 | 3.835 | 3.804 |
| Fe-I_2_ | 4.117 | 4.059 | 4.025 | 4.022 | 3.999 | 3.978 | 3.958 | 3.914 | 3.921 | 3.891 | 3.875 | 3.865 | 3.845 |
| Fe-I_3_ | 4.119 | 4.057 | 4.038 | 4.000 | 3.988 | 3.976 | 3.960 | 3.939 | 3.933 | 3.919 | 3.876 | 3.817 | 3.810 |
| Fe-I_4_ | 4.123 | 4.071 | 4.046 | 4.016 | 3.994 | 3.970 | 3.953 | 3.930 | 3.915 | 3.887 | 3.872 | 3.845 | 3.806 |
| Fe-I_5_ | 4.124 | 4.072 | 4.008 | 4.002 | 3.985 | 3.973 | 3.957 | 3.935 | 3.916 | 3.889 | 3.857 | 3.842 | 3.762 |
| Fe-I_6_ | 4.123 | 4.063 | 4.025 | 4.017 | 3.997 | 3.974 | 3.958 | 3.930 | 3.913 | 3.890 | 3.866 | 3.854 | 3.728 |
| Fe-C | 4.040 | 3.985 | 3.960 | 3.967 | 3.931 | 3.915 | 3.899 | 3.873 | 3.870 | 3.856 | 3.840 | 3.833 | 3.748 |

Table S4. Number of unpaired electrons (NUE) in Fe atoms of GR4x6 models, and relative differences in the NUE between outer, inner (two kinds of inner Fe, denoted by I and i) and central Fe atoms for the simulated GR4x6 models with cluster charge differing from +6 to -16. Δ1_O-I_ denotes the difference between minimum and maximum NUE of outer and inner (I, big cycle) Fe, respectively; Δ2_O-I_ denotes the difference between average NUEs of outer and inner (I, big cycle) Fe atoms, respectively; Δ1_I-i_ denotes the difference between minimum and maximum NUE of inner (I, big cycle) and inner (i, small cycle) Fe atoms, respectively; Δ2_I-i_ denotes the difference between average NUEs of inner (I, big cycle) and inner (i, small cycle) Fe atoms, respectively; Δ1_i-C_ denotes the difference between minimum and maximum NUE of inner (i, small cycle) and central Fe atoms, respectively; Δ2_i-C_ denotes the difference between average NUEs of inner (i, small cycle) and central Fe atoms, respectively.

|  | 6 | 5 | 4 | 3 | 2 | 1 | 0 | -1 | -2 | -3 | -4 | -5 |
| --- | --- | --- | --- | --- | --- | --- | --- | --- | --- | --- | --- | --- |
| Fe-O_1_ | 4.121 | 4.124 | 4.127 | 4.132 | 4.135 | 4.137 | 4.141 | 4.144 | 4.150 | 4.152 | 4.160 | 4.161 |
| Fe-O_2_ | 4.181 | 4.183 | 4.190 | 4.183 | 4.180 | 4.162 | 4.143 | 4.128 | 4.116 | 4.096 | 4.079 | 4.064 |
| Fe-O_3_ | 4.163 | 4.169 | 4.165 | 4.165 | 4.162 | 4.154 | 4.141 | 4.133 | 4.106 | 4.088 | 4.065 | 4.050 |
| Fe-O_4_ | 4.135 | 4.140 | 4.144 | 4.146 | 4.151 | 4.154 | 4.158 | 4.160 | 4.158 | 4.157 | 4.160 | 4.160 |
| Fe-O_5_ | 4.181 | 4.184 | 4.187 | 4.185 | 4.164 | 4.147 | 4.119 | 4.105 | 4.088 | 4.076 | 4.066 | 4.049 |
| Fe-O_6_ | 4.163 | 4.168 | 4.169 | 4.166 | 4.162 | 4.141 | 4.117 | 4.102 | 4.088 | 4.076 | 4.063 | 4.043 |
| Fe-O_7_ | 4.160 | 4.165 | 4.169 | 4.175 | 4.176 | 4.178 | 4.184 | 4.186 | 4.180 | 4.163 | 4.146 | 4.132 |
| Fe-O_8_ | 4.160 | 4.165 | 4.167 | 4.166 | 4.156 | 4.147 | 4.135 | 4.123 | 4.113 | 4.099 | 4.085 | 4.072 |
| Fe-O_9_ | 4.157 | 4.157 | 4.168 | 4.168 | 4.170 | 4.160 | 4.134 | 4.117 | 4.101 | 4.090 | 4.082 | 4.073 |
| Fe-O_10_ | 4.158 | 4.164 | 4.168 | 4.173 | 4.174 | 4.178 | 4.182 | 4.173 | 4.166 | 4.154 | 4.145 | 4.134 |
| Fe-O1_1_ | 4.158 | 4.168 | 4.164 | 4.163 | 4.161 | 4.145 | 4.126 | 4.122 | 4.104 | 4.098 | 4.083 | 4.067 |
| Fe-O_12_ | 4.188 | 4.192 | 4.186 | 4.176 | 4.158 | 4.142 | 4.123 | 4.114 | 4.099 | 4.091 | 4.073 | 4.062 |
| Fe-O_13_ | 4.134 | 4.140 | 4.146 | 4.149 | 4.155 | 4.157 | 4.161 | 4.158 | 4.158 | 4.155 | 4.157 | 4.149 |
| Fe-O_14_ | 4.151 | 4.156 | 4.160 | 4.159 | 4.157 | 4.156 | 4.150 | 4.133 | 4.113 | 4.102 | 4.094 | 4.080 |
| Fe-O_15_ | 4.150 | 4.158 | 4.169 | 4.172 | 4.158 | 4.145 | 4.139 | 4.131 | 4.113 | 4.103 | 4.094 | 4.084 |
| Fe-O_16_ | 4.159 | 4.164 | 4.169 | 4.173 | 4.172 | 4.160 | 4.154 | 4.150 | 4.145 | 4.139 | 4.131 | 4.120 |
| Fe-O_17_ | 4.175 | 4.176 | 4.178 | 4.177 | 4.163 | 4.149 | 4.140 | 4.123 | 4.104 | 4.086 | 4.049 | 4.036 |
| Fe-O_18_ | 4.184 | 4.187 | 4.180 | 4.175 | 4.164 | 4.157 | 4.140 | 4.126 | 4.105 | 4.084 | 4.054 | 4.040 |
| Fe-I_1_ | 4.131 | 4.102 | 4.072 | 4.051 | 4.036 | 4.017 | 4.009 | 4.005 | 3.994 | 3.980 | 3.960 | 3.951 |
| Fe-I_2_ | 4.087 | 4.059 | 4.046 | 4.024 | 4.011 | 3.999 | 3.986 | 3.973 | 3.962 | 3.952 | 3.948 | 3.940 |
| Fe-I_3_ | 4.155 | 4.117 | 4.079 | 4.056 | 4.032 | 4.019 | 4.016 | 4.006 | 3.996 | 3.983 | 3.972 | 3.958 |
| Fe-I_4_ | 4.085 | 4.059 | 4.052 | 4.033 | 4.012 | 3.999 | 3.988 | 3.979 | 3.972 | 3.961 | 3.951 | 3.922 |
| Fe-I_5_ | 4.135 | 4.115 | 4.089 | 4.069 | 4.044 | 4.030 | 4.023 | 4.014 | 3.999 | 3.986 | 3.981 | 3.966 |
| Fe-I_6_ | 4.115 | 4.075 | 4.068 | 4.039 | 4.019 | 4.003 | 3.993 | 3.985 | 3.979 | 3.967 | 3.953 | 3.939 |
| Fe-I_7_ | 4.153 | 4.118 | 4.086 | 4.062 | 4.041 | 4.029 | 4.019 | 4.005 | 4.004 | 3.997 | 3.982 | 3.973 |
| Fe-I_8_ | 4.092 | 4.086 | 4.044 | 4.029 | 4.010 | 3.996 | 3.985 | 3.982 | 3.978 | 3.971 | 3.957 | 3.955 |
| Fe-I_9_ | 4.157 | 4.124 | 4.082 | 4.057 | 4.044 | 4.030 | 4.019 | 4.010 | 4.002 | 3.989 | 3.983 | 3.969 |
| Fe-I_10_ | 4.119 | 4.084 | 4.055 | 4.032 | 4.011 | 4.012 | 3.998 | 3.978 | 3.969 | 3.966 | 3.959 | 3.957 |
| Fe-I_11_ | 4.152 | 4.135 | 4.113 | 4.068 | 4.052 | 4.016 | 4.017 | 4.010 | 3.994 | 3.987 | 3.989 | 3.982 |
| Fe-I_12_ | 4.095 | 4.060 | 4.033 | 4.024 | 4.004 | 4.002 | 3.985 | 3.972 | 3.960 | 3.941 | 3.923 | 3.909 |
| Fe-i_1_ | 4.032 | 4.005 | 3.981 | 3.969 | 3.952 | 3.952 | 3.937 | 3.927 | 3.912 | 3.905 | 3.886 | 3.868 |
| Fe-i_2_ | 4.016 | 4.009 | 3.983 | 3.966 | 3.950 | 3.954 | 3.943 | 3.938 | 3.911 | 3.910 | 3.899 | 3.888 |
| Fe-i_3_ | 4.014 | 4.006 | 3.977 | 3.963 | 3.951 | 3.948 | 3.930 | 3.919 | 3.918 | 3.908 | 3.893 | 3.870 |
| Fe-i_4_ | 4.023 | 4.003 | 3.993 | 3.981 | 3.953 | 3.945 | 3.932 | 3.920 | 3.915 | 3.907 | 3.890 | 3.891 |
| Fe-i_5_ | 4.047 | 4.010 | 4.002 | 3.979 | 3.966 | 3.958 | 3.946 | 3.935 | 3.925 | 3.920 | 3.912 | 3.899 |
| Fe-i_6_ | 4.032 | 4.010 | 3.999 | 3.970 | 3.954 | 3.949 | 3.937 | 3.922 | 3.934 | 3.921 | 3.903 | 3.896 |
| Fe-C | 3.991 | 3.980 | 3.963 | 3.951 | 3.947 | 3.947 | 3.922 | 3.916 | 3.912 | 3.897 | 3.886 | 3.874 |
| Δ1_O-I_ | -0.064 | -0.062 | -0.053 | -0.043 | -0.029 | -0.020 | -0.023 | -0.024 | -0.017 | -0.007 | -0.005 | -0.004 |
| Δ2_O-I_ | 0.028 | 0.058 | 0.086 | 0.108 | 0.122 | 0.128 | 0.128 | 0.129 | 0.128 | 0.128 | 0.128 | 0.128 |
| Δ1_I-i_ | -0.010 | -0.001 | 0.011 | 0.001 | 0.006 | -0.005 | -0.001 | 0.001 | 0.001 | 0.012 | 0.025 | 0.013 |
| Δ2_I-i_ | 0.093 | 0.090 | 0.085 | 0.079 | 0.078 | 0.067 | 0.072 | 0.073 | 0.071 | 0.069 | 0.073 | 0.074 |
| Δ1_i-C_ | 0.024 | 0.023 | 0.013 | 0.012 | 0.003 | -0.002 | 0.008 | 0.003 | -0.001 | 0.009 | 0.000 | -0.005 |
| Δ2_i-C_ | 0.046 | 0.035 | 0.032 | 0.028 | 0.015 | 0.011 | 0.022 | 0.018 | 0.013 | 0.019 | 0.015 | 0.015 |

Table S4-continued

|  | -6 | -7 | -8 | -9 | -10 | -11 | -12 | -13 | -14 | -15 | -16 |
| --- | --- | --- | --- | --- | --- | --- | --- | --- | --- | --- | --- |
| Fe-O_1_ | 4.155 | 4.152 | 4.135 | 4.106 | 4.098 | 4.072 | 4.067 | 4.039 | 4.030 | 3.995 | 3.987 |
| Fe-O_2_ | 4.040 | 4.028 | 4.021 | 4.010 | 4.000 | 3.989 | 3.980 | 3.964 | 3.956 | 3.935 | 3.928 |
| Fe-O_3_ | 4.052 | 4.036 | 4.038 | 4.034 | 4.016 | 4.002 | 3.995 | 3.964 | 3.955 | 3.928 | 3.935 |
| Fe-O_4_ | 4.132 | 4.127 | 4.100 | 4.054 | 4.048 | 4.032 | 4.038 | 3.984 | 3.967 | 3.950 | 3.932 |
| Fe-O_5_ | 4.044 | 4.032 | 4.018 | 3.994 | 3.987 | 3.980 | 3.964 | 3.968 | 3.946 | 3.932 | 3.919 |
| Fe-O_6_ | 4.066 | 4.048 | 4.050 | 4.041 | 4.032 | 4.017 | 3.967 | 3.974 | 3.956 | 3.932 | 3.935 |
| Fe-O_7_ | 4.101 | 4.095 | 4.063 | 4.030 | 4.021 | 4.014 | 4.012 | 3.987 | 3.960 | 3.935 | 3.930 |
| Fe-O_8_ | 4.069 | 4.063 | 4.041 | 4.015 | 4.011 | 4.009 | 3.997 | 3.984 | 3.954 | 3.936 | 3.930 |
| Fe-O_9_ | 4.068 | 4.059 | 4.045 | 4.033 | 4.015 | 3.996 | 3.976 | 3.983 | 3.942 | 3.916 | 3.910 |
| Fe-O_10_ | 4.110 | 4.096 | 4.079 | 4.048 | 4.029 | 4.013 | 4.014 | 3.987 | 3.954 | 3.918 | 3.865 |
| Fe-O1_1_ | 4.049 | 4.021 | 4.021 | 4.020 | 4.027 | 4.013 | 3.976 | 3.983 | 3.942 | 3.916 | 3.910 |
| Fe-O_12_ | 4.040 | 4.020 | 4.017 | 4.011 | 4.003 | 3.987 | 3.967 | 3.972 | 3.930 | 3.932 | 3.897 |
| Fe-O_13_ | 4.147 | 4.136 | 4.128 | 4.110 | 4.084 | 4.062 | 4.047 | 4.013 | 3.982 | 3.964 | 3.929 |
| Fe-O_14_ | 4.060 | 4.040 | 4.023 | 4.020 | 4.008 | 4.002 | 3.995 | 3.973 | 3.953 | 3.925 | 3.919 |
| Fe-O_15_ | 4.063 | 4.043 | 4.023 | 4.016 | 4.006 | 3.998 | 3.993 | 3.977 | 3.968 | 3.948 | 3.922 |
| Fe-O_16_ | 4.109 | 4.103 | 4.096 | 4.088 | 4.065 | 4.049 | 4.020 | 3.985 | 3.960 | 3.938 | 3.936 |
| Fe-O_17_ | 4.027 | 4.019 | 4.014 | 4.010 | 3.991 | 3.974 | 3.978 | 3.962 | 3.936 | 3.916 | 3.882 |
| Fe-O_18_ | 4.030 | 4.021 | 4.018 | 4.018 | 3.996 | 3.981 | 3.978 | 3.967 | 3.948 | 3.931 | 3.910 |
| Fe-I_1_ | 3.945 | 3.934 | 3.922 | 3.918 | 3.887 | 3.875 | 3.854 | 3.865 | 3.860 | 3.853 | 3.849 |
| Fe-I_2_ | 3.929 | 3.917 | 3.906 | 3.888 | 3.875 | 3.869 | 3.890 | 3.848 | 3.844 | 3.832 | 3.828 |
| Fe-I_3_ | 3.957 | 3.942 | 3.928 | 3.910 | 3.900 | 3.886 | 3.907 | 3.858 | 3.850 | 3.840 | 3.708 |
| Fe-I_4_ | 3.933 | 3.927 | 3.906 | 3.888 | 3.884 | 3.870 | 3.857 | 3.845 | 3.837 | 3.830 | 3.700 |
| Fe-I_5_ | 3.956 | 3.960 | 3.942 | 3.919 | 3.909 | 3.888 | 3.883 | 3.866 | 3.845 | 3.831 | 3.837 |
| Fe-I_6_ | 3.924 | 3.920 | 3.913 | 3.900 | 3.877 | 3.871 | 3.869 | 3.842 | 3.831 | 3.820 | 3.810 |
| Fe-I_7_ | 3.952 | 3.938 | 3.919 | 3.911 | 3.897 | 3.872 | 3.852 | 3.850 | 3.846 | 3.838 | 3.830 |
| Fe-I_8_ | 3.925 | 3.914 | 3.888 | 3.879 | 3.867 | 3.855 | 3.839 | 3.845 | 3.834 | 3.829 | 3.814 |
| Fe-I_9_ | 3.957 | 3.945 | 3.938 | 3.927 | 3.922 | 3.902 | 3.895 | 3.873 | 3.840 | 3.834 | 3.806 |
| Fe-I_10_ | 3.937 | 3.926 | 3.905 | 3.893 | 3.863 | 3.853 | 3.854 | 3.843 | 3.844 | 3.830 | 3.824 |
| Fe-I_11_ | 3.971 | 3.969 | 3.963 | 3.958 | 3.933 | 3.929 | 3.903 | 3.867 | 3.844 | 3.824 | 3.825 |
| Fe-I_12_ | 3.894 | 3.878 | 3.862 | 3.848 | 3.853 | 3.832 | 3.797 | 3.842 | 3.839 | 3.831 | 3.809 |
| Fe-i_1_ | 3.863 | 3.832 | 3.833 | 3.835 | 3.838 | 3.822 | 3.797 | 3.815 | 3.811 | 3.803 | 3.811 |
| Fe-i_2_ | 3.865 | 3.851 | 3.859 | 3.853 | 3.839 | 3.825 | 3.735 | 3.809 | 3.804 | 3.798 | 3.682 |
| Fe-i_3_ | 3.873 | 3.866 | 3.841 | 3.848 | 3.846 | 3.817 | 3.722 | 3.811 | 3.808 | 3.798 | 3.793 |
| Fe-i_4_ | 3.877 | 3.869 | 3.835 | 3.825 | 3.829 | 3.829 | 3.824 | 3.817 | 3.817 | 3.810 | 3.811 |
| Fe-i_5_ | 3.894 | 3.880 | 3.870 | 3.858 | 3.842 | 3.844 | 3.850 | 3.829 | 3.820 | 3.812 | 3.803 |
| Fe-i_6_ | 3.880 | 3.861 | 3.837 | 3.839 | 3.846 | 3.842 | 3.839 | 3.827 | 3.821 | 3.812 | 3.797 |
| Fe-C | 3.847 | 3.835 | 3.821 | 3.817 | 3.827 | 3.822 | 3.720 | 3.808 | 3.806 | 3.798 | 3.804 |
| Δ1_O-I_ | -0.003 | -0.002 | -0.003 | -0.025 | -0.009 | -0.007 | -0.014 | -0.004 | -0.018 | -0.015 | -0.046 |
| Δ2_O-I_ | 0.127 | 0.123 | 0.125 | 0.120 | 0.125 | 0.125 | 0.117 | 0.118 | 0.107 | 0.095 | 0.110 |
| Δ1_I-i_ | 0.030 | 0.034 | 0.019 | 0.021 | 0.010 | 0.009 | -0.011 | 0.000 | -0.009 | -0.011 | -0.111 |
| Δ2_I-i_ | 0.073 | 0.080 | 0.081 | 0.074 | 0.059 | 0.058 | 0.087 | 0.043 | 0.035 | 0.032 | 0.025 |
| Δ1_i-C_ | 0.016 | -0.003 | 0.012 | 0.008 | 0.001 | -0.006 | 0.002 | 0.000 | -0.002 | 0.000 | -0.122 |





# Figure S3. Spin density distribution among Fe atoms of GR2x6(-8) calculating using different DFT functionals.





# Figure S4. Spin density distribution among iron atoms of GR2x6(-8) with MP2 method.


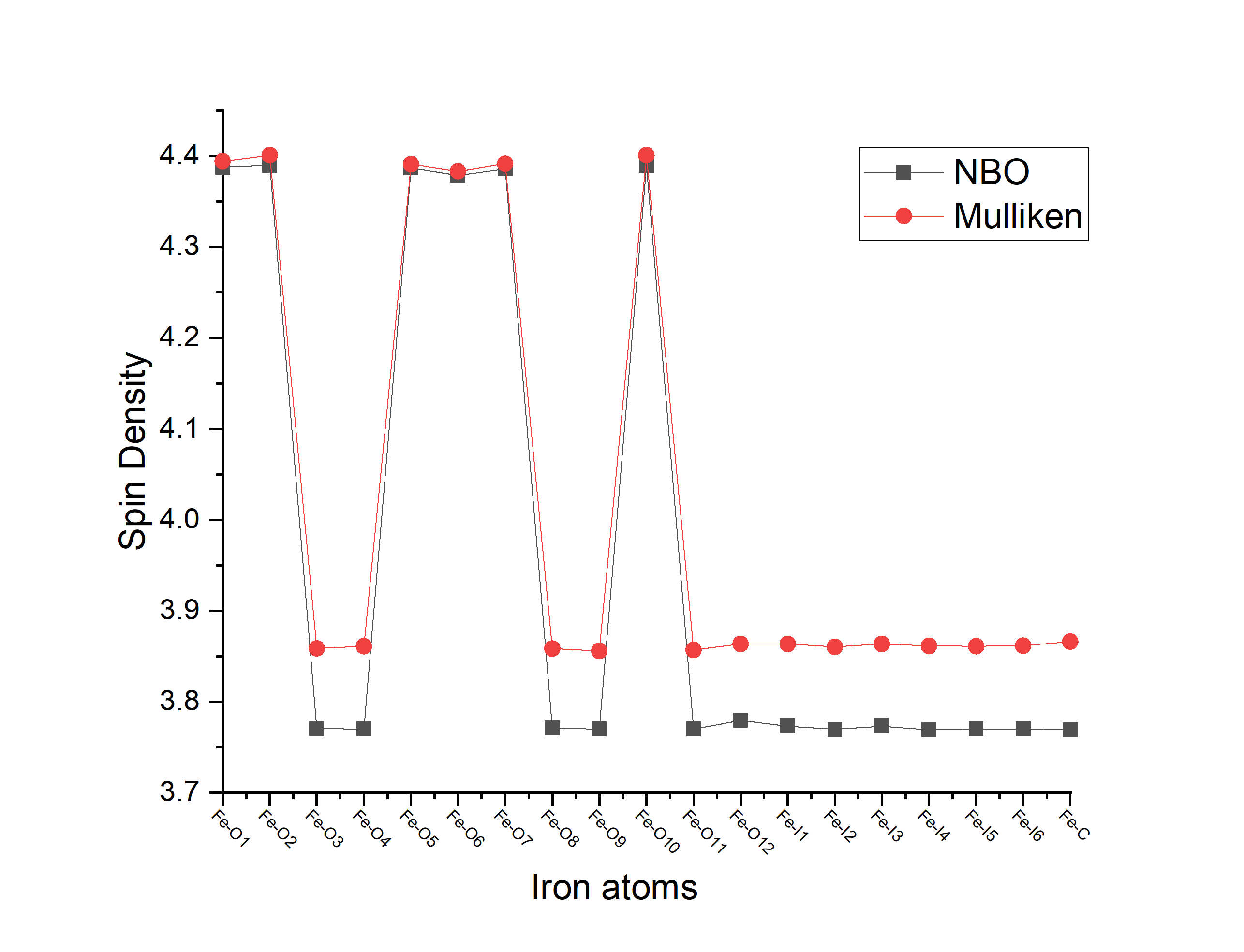


# Figure S5. Spin density distribution of Fe atoms in GR3x6(-10) with PBE0 functional.

Text S2. Effect of dielectric constant on spin density of Fe atoms

In this work, we only considered the GR sheet structure, which means that water molecules are in an open environment instead of a “channel”, hence we consider the infinite dielectric constant would be a reasonable choice in this work. Another reason for choosing an infinite dielectric constant is that the implicit solvent model COSMO-RS uses this as the reference state, regardless of the actual dielectric constant of the solvent, including for organic solvents. Predicted thermodynamics properties are reliable for water and organic solvents in the COSMO-RS method. However, we ran some calculations to see how a reduction of dielectric constant affected the spin density of Fe cations. We choose three representative models (GR2x6(-8), GR3x6(-10) and GR4x6(-10)) and ran the optimization and Mulliken analysis with dielectric constant ε=10. For GR2x6(-8), when the dielectric constant changed from infinite to 10, the spin density changed by maximum 0.134 (3.45%), which is a significant but small change. This slightly altered the spin density tendency such that the center Fe atom was no longer the most reductive one. However, the results from the decrease om the dielectric constant in the case of GR3x6(-10) and GR4x6(-10) showed that the spin density of each Fe cation basically remained the same (0.55% at most) and the spin density tendencies were practically identical for the two different dielectric constants (Figure S6, below). Since the medium and large sized GR models are more reliable than the small cluster, we can conclude that the dielectric constant has an insignificant effect on the absolute values of spin densities of Fe atoms, and it has no effect on the spin density distribution tendency of Fe cations.”

Table S5. The Mulliken unpaired electrons numbers of Fe atoms from GR2x6(-8) with different dielectric constants ε=10 and ε=infinite.

|  | Fe-O_1_ | Fe-O_2_ | Fe-O_3_ | Fe-O_4_ | Fe-O_5_ | Fe-O_6_ | Fe-C |
| --- | --- | --- | --- | --- | --- | --- | --- |
| ε=10 | 3.97 | 3.94 | 3.93 | 3.96 | 3.76 | 3.93 | 3.88 |
| ε=Infinite | 3.97 | 3.93 | 3.96 | 3.93 | 3.90 | 3.88 | 3.83 |
| Δ-Mulliken | -0.002 | -0.017 | 0.032 | -0.027 | 0.134 | -0.045 | -0.054 |
| Δ-Percentage | -0.05% | -0.43% | 0.81% | -0.67% | 3.45% | -1.17% | -1.40% |

Table S6. The Mulliken unpaired electrons numbers of Fe atoms from GR3x6(-10) with different dielectric constants ε=10 and ε=infinite.

|  | Fe-O_1_ | Fe-O_2_ | Fe-O_3_ | Fe-O_4_ | Fe-O_5_ | Fe-O_6_ | Fe-O_7_ | Fe-O_8_ | Fe-O_9_ | Fe-O_10_ |
| --- | --- | --- | --- | --- | --- | --- | --- | --- | --- | --- |
| ε=10 | 4.00 | 4.00 | 4.00 | 3.99 | 4.00 | 4.00 | 3.99 | 3.99 | 3.98 | 3.99 |
| ε=Infinite | 4.00 | 4.00 | 4.00 | 3.99 | 3.99 | 3.99 | 3.99 | 3.99 | 3.98 | 3.98 |
| Δ-Mulliken | -0.001 | -0.006 | -0.002 | 0.000 | -0.002 | -0.014 | -0.004 | -0.002 | -0.005 | -0.016 |
| Δ(Percentage) | -0.03% | -0.15% | -0.04% | 0.01% | -0.04% | -0.36% | -0.09% | -0.04% | -0.12% | -0.40% |
|  | Fe-O_11_ | Fe-O_12_ | Fe-I_1_ | Fe-I_2_ | Fe-I_3_ | Fe-I_4_ | Fe-I_5_ | Fe-I_6_ | Fe-C |  |
| ε=10 | 3.98 | 3.96 | 3.87 | 3.87 | 3.87 | 3.87 | 3.86 | 3.86 | 3.83 |  |
| ε=Infinite | 3.98 | 3.96 | 3.88 | 3.87 | 3.87 | 3.87 | 3.87 | 3.86 | 3.84 |  |
| Δ-Mulliken | -0.004 | -0.005 | 0.004 | 0.006 | 0.002 | 0.006 | 0.004 | -0.003 | 0.006 |  |
| Δ(Percentage) | -0.11% | -0.13% | 0.09% | 0.15% | 0.06% | 0.15% | 0.09% | -0.07% | 0.16% |  |

Table S7. The Mulliken unpaired electrons numbers of Fe atoms from GR4x6(-10) with different dielectric constants ε=10 and ε=infinite.

|  | Fe-O_1_ | Fe-O_2_ | Fe-O_3_ | Fe-O_4_ | Fe-O_5_ | Fe-O_6_ | Fe-O_7_ | Fe-O_8_ | Fe-O_9_ | Fe-O_10_ |
| --- | --- | --- | --- | --- | --- | --- | --- | --- | --- | --- |
| ε=10 | 4.12 | 4.10 | 4.06 | 4.03 | 4.07 | 4.02 | 4.01 | 4.02 | 4.04 | 4.01 |
| ε=Infinite | 4.10 | 4.08 | 4.05 | 4.03 | 4.07 | 4.02 | 4.01 | 4.01 | 4.03 | 4.01 |
| Δ-Mulliken | -0.020 | -0.014 | -0.012 | -0.006 | -0.007 | -0.003 | 0.002 | -0.005 | -0.006 | 0.000 |
| Δ(Percentage) | -0.49% | -0.35% | -0.29% | -0.14% | -0.18% | -0.06% | 0.04% | -0.11% | -0.14% | 0.00% |
|  | Fe-O_11_ | Fe-O_12_ | Fe-O_13_ | Fe-O_14_ | Fe-O_15_ | Fe-O_16_ | Fe-O_17_ | Fe-O_18_ | Fe-I_1_ | Fe-I_2_ |
| ε=10 | 4.01 | 4.02 | 4.03 | 3.99 | 4.00 | 4.01 | 4.01 | 4.00 | 3.93 | 3.92 |
| ε=Infinite | 4.01 | 4.02 | 4.03 | 3.99 | 4.00 | 4.00 | 4.00 | 3.99 | 3.93 | 3.92 |
| Δ-Mulliken | -0.001 | -0.008 | 0.000 | -0.004 | 0.001 | -0.008 | -0.011 | -0.004 | 0.002 | 0.006 |
| Δ(Percentage) | -0.03% | -0.21% | -0.01% | -0.09% | 0.03% | -0.20% | -0.28% | -0.11% | 0.06% | 0.14% |
|  | Fe-I_3_ | Fe-I_4_ | Fe-I_5_ | Fe-I_6_ | Fe-I_7_ | Fe-I_8_ | Fe-I_9_ | Fe-I_10_ | Fe-I_11_ | Fe-I_12_ |
| ε=10 | 3.90 | 3.90 | 3.89 | 3.89 | 3.86 | 3.87 | 3.87 | 3.86 | 3.87 | 3.85 |
| ε=Infinite | 3.90 | 3.91 | 3.90 | 3.89 | 3.86 | 3.88 | 3.87 | 3.87 | 3.88 | 3.85 |
| Δ-Mulliken | 0.001 | 0.006 | 0.002 | 0.001 | 0.003 | 0.012 | 0.006 | 0.005 | 0.011 | 0.004 |
| Δ(Percentage) | 0.03% | 0.14% | 0.06% | 0.03% | 0.09% | 0.30% | 0.14% | 0.14% | 0.27% | 0.10% |
|  | Fe-i_1_ | Fe-i_2_ | Fe-i_3_ | Fe-i_4_ | Fe-i_5_ | Fe-i_6_ | Fe-C |  |  |  |
| ε=10 | 3.83 | 3.83 | 3.82 | 3.83 | 3.82 | 3.82 | 3.81 |  |  |  |
| ε=Infinite | 3.84 | 3.85 | 3.83 | 3.85 | 3.84 | 3.84 | 3.83 |  |  |  |
| Δ-Mulliken | 0.014 | 0.016 | 0.011 | 0.016 | 0.015 | 0.017 | 0.021 |  |  |  |
| Δ(Percentage) | 0.37% | 0.41% | 0.28% | 0.42% | 0.38% | 0.43% | 0.55% |  |  |  |





Figure S6. In a) GR2x6(-8), b) GR3x6(-10) and c) GR4x6(-10), the comparison of Mulliken unpaired electrons numbers of each Fe atoms with different dielectric constant environment, ε=10 and ε=infinite.

Text S3. Magnetism discussion

The total magnetic moment in the Russel-Saunders scheme is defined as

$$\mu=g_{e}\sqrt{S\left( S+1 \right)}\mu_{B}$$

where g_e_ is the gyromagnetic ratio, normally taken to be the approximated value that is 2, S is the total spin moment, and μ_B_ is the Bohr magneton. Hence, in high-spin Fe cations, the theoretical total magnetic moments are 4.90μ_B_ for Fe(II) and 5.92μ_B_ for Fe(III). In this work, we calculated the d-orbital magnetic moment of each Fe cation for a few selected representative models (below, Table S8-S11) and analysed the GR magnetic properties.

First, we can see from the table that, similar to spin density distribution tendency, from interior to exterior of GR sheet, the magnetic moments of Fe atoms generally increase, which make sense because the magnetic moments are contributed to unpaired electrons. Second, for all the Fe atoms, the magnetic moments are smaller than the theoretical value of Fe(II), i.e., 4.9μ_B_, one of the reasons might be the electron’s delocalization from Fe s- and p-orbital, and surrounding H and O atoms to Fe d-orbital.

To calculate the total magnetic moments, we extracted the calculated S*S values of the GR model, then applied the above equation to calculate the magnetic moments. The theoretical total magnetic moments were calculated by the theoretical S value, which is determined by the applied unpaired electrons. The results show that for all the selected models, the calculated magnetic moments are slightly higher that the theoretical ones. The results could indicate that all the Fe spins are aligned paralleled, which is consistent our findings that the highest possible total spin is the most stable spin state, which indicates a ferromagnetic single GR sheet. As this is a single GR sheet study, this cannot tell us the overall material magnetic properties. There could be alternating spins between different layers in green rust, which would make green rust a ferrimagnetic material, unless the spins alternated every other layer, which would make GR antiferromagnetic. Moreover, the results also show that different DFT functionals all show consistent magnetic properties in our model.

Table S8. The magnetic moment of each Fe atom and calculated and theoretical total magnetic moment of GR2x6(-6) and GR2x6(-8).

|  | Fe-O_1_ | Fe-O_2_ | Fe-O_3_ | Fe-O_4_ | Fe-O_5_ | Fe-O_6_ | Fe-C | Total_Calc. | Total_Theor. |
| --- | --- | --- | --- | --- | --- | --- | --- | --- | --- |
| GR2x6(-6), μ_B_ | 4.82 | 4.82 | 4.79 | 4.75 | 4.74 | 4.74 | 4.70 | 33.96 | 32.98 |
| GR2x6(-8), μ_B_ | 4.71 | 4.68 | 4.71 | 4.68 | 4.65 | 4.64 | 4.61 | 31.96 | 30.98 |

Table S9. The magnetic moment of each Fe atoms and calculated total magnetic moment of GR2x6(-8) with different functionals.

|  | Fe-O_1_ | Fe-O_2_ | Fe-O_3_ | Fe-O_4_ | Fe-O_5_ | Fe-O_6_ | Fe-C | Total_Calc. |
| --- | --- | --- | --- | --- | --- | --- | --- | --- |
| BLYP | 4.71 | 4.69 | 4.71 | 4.69 | 4.65 | 4.66 | 4.63 | 31.96 |
| PBE | 4.71 | 4.69 | 4.71 | 4.69 | 4.65 | 4.64 | 4.61 | 31.97 |
| TPSS | 4.75 | 4.70 | 4.73 | 4.71 | 4.65 | 4.68 | 4.63 | 31.97 |
| B3LYP | 5.03 | 4.63 | 5.03 | 4.63 | 4.63 | 4.63 | 4.64 | 31.97 |
| TPSSh | 4.89 | 4.65 | 4.88 | 4.75 | 4.62 | 4.74 | 4.64 | 31.97 |
| PBE0 | 5.07 | 4.64 | 5.07 | 4.63 | 4.64 | 4.64 | 4.65 | 31.97 |

Table S10. The magnetic moment of each Fe atoms and calculated and theoretical total magnetic moment of GR3x6(-7) and GR3x6(-10).

|  | Fe-O_1_ | Fe-O_2_ | Fe-O_3_ | Fe-O_4_ | Fe-O_5_ | Fe-O_6_ | Fe-O_7_ | Fe-O_8_ | Fe-O_9_ | Fe-O_10_ | Fe-O_11_ |
| --- | --- | --- | --- | --- | --- | --- | --- | --- | --- | --- | --- |
| GR3x6(-7), μ_B_ | 4.77 | 4.82 | 4.75 | 4.76 | 4.79 | 4.79 | 4.75 | 4.77 | 4.74 | 4.82 | 4.75 |
| GR3x6(-10), μ_B_ | 4.71 | 4.70 | 4.72 | 4.72 | 4.71 | 4.72 | 4.71 | 4.72 | 4.69 | 4.72 | 4.73 |
|  | Fe-O_12_ | Fe-I_1_ | Fe-I_2_ | Fe-I_3_ | Fe-I_4_ | Fe-I_5_ | Fe-I_6_ | Fe-C | Total-Calc. | Total_Theor. |  |
| GR3x6(-7), μ_B_ | 4.76 | 4.67 | 4.66 | 4.67 | 4.66 | 4.67 | 4.66 | 4.62 | 86.99 | 85.99 |  |
| GR3x6(-10), μ_B_ | 4.72 | 4.63 | 4.63 | 4.63 | 4.63 | 4.62 | 4.62 | 4.60 | 83.99 | 82.99 |  |

Table S11. The magnetic moment of each Fe atoms and calculated and theoretical total magnetic moment of GR4x6(-7) and GR4x6(-10).

|  | Fe-O_1_ | Fe-O_2_ | Fe-O_3_ | Fe-O_4_ | Fe-O_5_ | Fe-O_6_ | Fe-O_7_ | Fe-O_8_ | Fe-O_9_ | Fe-O_10_ |
| --- | --- | --- | --- | --- | --- | --- | --- | --- | --- | --- |
| GR4x6(-7), μ_B_ | 4.85 | 4.83 | 4.83 | 4.79 | 4.80 | 4.79 | 4.77 | 4.77 | 4.75 | 4.76 |
| GR3x6(-10), μ_B_ | 4.81 | 4.79 | 4.77 | 4.74 | 4.77 | 4.74 | 4.74 | 4.74 | 4.74 | 4.73 |
|  | Fe-O_11_ | Fe-O_12_ | Fe-O_13_ | Fe-O_14_ | Fe-O_15_ | Fe-O_16_ | Fe-O_17_ | Fe-O_18_ | Fe-I_1_ | Fe-I_2_ |
| GR3x6(-7), μ_B_ | 4.76 | 4.75 | 4.74 | 4.74 | 4.74 | 4.74 | 4.74 | 4.74 | 4.69 | 4.68 |
| GR3x6(-10), μ_B_ | 4.73 | 4.73 | 4.75 | 4.71 | 4.73 | 4.72 | 4.72 | 4.72 | 4.67 | 4.66 |
|  | Fe-I_3_ | Fe-I_4_ | Fe-I_5_ | Fe-I_6_ | Fe-I_7_ | Fe-I_8_ | Fe-I_9_ | Fe-I_10_ | Fe-I_11_ | Fe-I_12_ |
| GR3x6(-7), μ_B_ | 4.68 | 4.69 | 4.67 | 4.67 | 4.66 | 4.66 | 4.66 | 4.66 | 4.66 | 4.64 |
| GR3x6(-10), μ_B_ | 4.65 | 4.65 | 4.64 | 4.64 | 4.62 | 4.63 | 4.63 | 4.63 | 4.63 | 4.61 |
|  | Fe-i_1_ | Fe-i_2_ | Fe-i_3_ | Fe-i_4_ | Fe-i_5_ | Fe-i_6_ | Fe-C | Total_Calc. | Total_Theor. |  |
| GR3x6(-7), μ_B_ | 4.63 | 4.62 | 4.62 | 4.62 | 4.61 | 4.60 | 4.60 | 164.99 | 163.99 |  |
| GR3x6(-10), μ_B_ | 4.60 | 4.61 | 4.59 | 4.60 | 4.60 | 4.60 | 4.60 | 161.99 | 160.99 |  |
